# Not only an inhibitor: Trehalose enhances the catalytic action exerted on oxaloacetate by rabbit lactate dehydrogenase

**DOI:** 10.1002/pro.70304

**Published:** 2025-09-13

**Authors:** Alessandra Stefan, Alejandro Hochkoeppler

**Affiliations:** ^1^ Department of Pharmacy and Biotechnology University of Bologna Bologna Italy; ^2^ CSGI, University of Firenze Sesto Fiorentino Italy

**Keywords:** lactate dehydrogenase, oxaloacetate, pyruvate, stopped‐flow, trehalose

## Abstract

Trehalose is an osmolyte featuring a prominent competence in stabilizing proteins and enzymes. In particular, in the presence of this peculiar disaccharide, quite a number of enzymes show improved stability against thermal denaturation and an enhanced ability to withstand exposure to freezing–thawing cycles. Moreover, it was recently reported that trehalose counteracts the acid‐induced dissociation of oligomeric protein complexes. Concerning the catalytic action of enzymes, the addition of trehalose to assay mixtures was found to decrease the values of both *K*_m_ and *k*_cat_, with the decrease in reaction velocity related to the increase in viscosity induced by the disaccharide. Here, we show that trehalose does not necessarily perform as a negative effector on reaction velocity. Using tetrameric rabbit muscle lactate dehydrogenase (rbLDH) as a model system, we report that trehalose does highly stimulate the catalytic action of this enzyme at the expense of oxaloacetate. In particular, stopped‐flow assays revealed that trehalose slows down the binding of *β*‐NADH to rbLDH, as well as its dissociation from the cofactor‐enzyme complex. Conversely, the presence of the disaccharide does not alter the rate constant of the association to rbLDH of the substrate analogue oxamate. Furthermore, according to steady‐state and stopped‐flow assays, we present evidence that the increased velocity of oxaloacetate reduction triggered by trehalose is related to an improved occupancy by the substrate of the enzyme subunits, and by a favorable reciprocal orientation of *β*‐NADH and oxaloacetate.

## 1 INTRODUCTION

When facing environmental conditions triggering an osmotic stress, both prokaryotic and eukaryotic cells are induced to undertake the synthesis of osmolytes (Yancey et al., 1982). Interestingly, the repertoire of these compounds contains chemically unrelated members, for example, polyols, amino acids, and substituted amines. In particular, osmolytes can be sub‐grouped into two sets, that is, compatible and counteracting solutes. The solutes defined as compatible do not interfere with cellular metabolism, albeit they are known to inhibit the catalytic action of enzymes (Borowitzka & Brown, 1974). Furthermore, compounds capable of antagonizing the harmful effects triggered by some osmolytes (e.g., urea) are denoted as counteracting solutes. Among compatible solutes, glycerol is a well‐known example (Borowitzka & Brown, 1974), and trimethylamine N‐oxide (TMAO) embodies a counteracting solute competent in antagonizing the effects induced by urea on proteins (Yancey & Somero, 1979).

The disaccharide trehalose (*α*‐D‐glucopyranosyl‐(1 → 1)‐*α*‐D‐glucopyranoside) is a compatible solute known to stabilize proteins and enzymes under low‐water conditions (Adler & Lee, 1999; Kreilgaard et al., 1999; Uritani et al., 1995). Moreover, it was reported that trehalose is effective in preventing the decrease of the activity of enzymes exposed to freeze‐drying procedures (Sampedro et al., 1998). In addition, trehalose can be conveniently used to investigate, at room temperature, the conformational transitions associated with the reaction steps catalyzed by an enzyme. In particular, this was shown for bacterial reaction centers embedded in trehalose glasses featuring different levels of dehydration (Malferrari et al., 2015; Nalepa et al., 2017).

Besides its competence in stabilizing proteins embedded in low‐water systems, trehalose is capable of preserving enzymes in solution. Trehalose was indeed reported to improve the stability of enzymes subjected to aqueous solutions under denaturing conditions induced by high temperatures, chaotropic agents, and freeze–thaw cycles. In particular, trehalose was shown to increase the melting temperature (*T*_m_) of bovine pancreatic RNase A, cutinase, and chicken lysozyme (Baptista et al., 2000; Kaushik & Bhat, 2003; Lin & Timasheff, 1996). Moreover, it was observed that the addition of 0.6 M trehalose to reaction mixtures stabilizes against thermal inactivation bovine pancreatic DNase I and the *Nco*I restriction endonuclease (Carninci et al., 1998). Surprisingly enough, it was also shown that trehalose counteracts the denaturation of *α*‐chymotrypsin induced by urea. In particular, in the presence of a disaccharide/chaotrope molar ratio equal to 0.5, a complete suppression of the effects triggered by urea alone was detected (Kumar et al., 2010). The storage of frozen enzymes is frequently associated with the loss of activity occurring when the icy enzymes are thawed. Experiments performed with bacterial *β*‐galactosidase revealed that the enzyme lost more than 80% of its catalytic action when subjected to freezing at −20°C for 72 h. However, no loss of activity was observed when 0.29 M trehalose was added to the *β*‐galactosidase solution before its freezing (Mitchell et al., 2019).

The functional properties of proteins depend on a multiplicity of factors, the majority of which can be affected by trehalose. First, trehalose slows down the dynamics of the water molecules interacting with the surface of proteins (i.e., the hydration water) (Fedorov et al., 2011; Corradini et al., 2013; Giuffrida et al., 2013; Ghatty & Carri, 2013; Fogarty & Laage, 2014; Schirò et al., 2015), and it should be noted that this interaction was found to be essential for the exertion of protein functions (Camisasca et al., 2023; Schirò et al., 2015). Furthermore, taking into account that the conformational dynamics of a protein are coupled to the reorganization of hydration water (Bellissent‐Funel et al., 2016; Schirò et al., 2015; Zhang et al., 2007), trehalose is recognized as capable of acting on the frequency of proteins' structural fluctuations. Notably, this action is not exerted by trehalose via its direct interaction with the surface of proteins. Trehalose is indeed responsible for the preferential hydration of proteins, that is, the disaccharide is excluded from the surface of proteins, where water is instead located (Lins et al., 2004; Timasheff, 2002). Importantly enough, the competence of trehalose in affecting the conformational dynamics of proteins translates into its capability to act on the catalytic performance of enzymes. It was indeed reported for quite a number of enzymes that the exertion of catalysis relies on conformational fluctuations (Palmer, 2004; Rozovsky et al., 2001; McElheny et al., 2005; Ishima et al., 1999; Cole & Loria, 2002; Duff Jr. et al., 2018), the amplitude of which can extend to the reshaping of secondary structure elements and to the mutual reorientation of domains (Bermek et al., 2011; Li et al., 1998). Interestingly, it has been shown that the structural elements of enzymes featuring dynamic rearrangements undergo concerted movements according to rate constants whose values are comparable to those of the corresponding catalytic rate constant, *k*_cat_ (Agarwal et al., 2004; Eisenmesser et al., 2005). Quite intriguingly, the link between conformational dynamics and catalytic action was extended to interpret allostery. It was indeed proposed that the occurrence in enzymes of conformational motions about a mean position represents per se a major determinant of allosteric transitions (Cooper & Dryden, 1984).

Among the factors affecting the velocity of reactions and therefore their rate constants, viscosity is an important determinant. Accordingly, quite a number of years ago, Hendrik Kramers introduced the viscosity of the medium as an additional factor to the pre‐exponential term of the Arrhenius equation (Kramers, 1940). In particular, when applied to enzyme‐catalyzed reactions, Kramers's equation implies that *k*_cat_ is inversely proportional to the viscosity of the medium. In addition to experiments aiming at a quantitative determination of this dependence (Doshi et al., 2012; Gavish & Werber, 1979), Kramers's theory has been used to interpret the effect of medium viscosity on ligand binding by proteins (Beece et al., 1980). Among the compounds used to alter the viscosity of reaction mixtures, glycerol, sucrose, and trehalose are usually selected. In particular, the activity of different enzymes was shown to be inhibited in the presence of trehalose concentrations raising the medium viscosity (Hernández‐Meza & Sampedro, 2018; Sampedro et al., 2002; Sampedro & Uribe, 2004; Uribe & Sampedro, 2003).

Lactate dehydrogenases (LDHs) catalyze the reversible reduction of pyruvate to lactate, with *β*‐NADH/*β*‐NAD^+^ or FADH_2_/FAD as the cofactor engaged by the enzyme in the redox reaction (Everse & Kaplan, 1973; Garvie, 1980). LDHs are oligomeric enzymes featuring dimeric or tetrameric quaternary structures, and their action is strictly enantioselective, leading to the generation of L‐ or D‐lactate. Among the L‐LDHs of vertebrates, two major isoforms were long ago identified, that is, the heart (H, LDH‐B) and the muscle (M, LDH‐A) enzyme (Kaplan et al., 1956, 1960). The rabbit skeletal muscle LDH is a tetrameric NADH‐dependent enzyme composed of four identical LDH‐A subunits, whose action is specific for the generation of L‐lactate. This catalytic action is exerted according to an ordered mechanism, that is, binding the redox cofactor first and only subsequently pyruvate, lactate, or the competitive inhibitor oxamate. Quite recently, the stabilization by trehalose of rabbit LDH‐A subjected to freeze‐drying was inspected (Kawai & Suzuki, 2007). Interestingly, the stabilization of the enzyme provided by trehalose was found to outperform those exerted by sucrose, maltose, and lactose (Kawai & Suzuki, 2007). Surprisingly enough, trehalose was also found to enhance the immobilization yield of rabbit LDH‐A and to counteract the dissociation of the enzyme induced by acidic pH conditions (Jackson et al., 2016; Simongini et al., 2023). In addition, the effect of trehalose on rabbit LDH‐A kinetics was investigated. Performing steady‐state activity assays at pH 7 in the absence or in the presence of trehalose, it was shown that trehalose lowers the *K*_m_ for pyruvate and decreases *k*_cat_ (Hernández‐Meza & Sampedro, 2018). We have subsequently extended these observations by determining the activity of rabbit LDH‐A as a function of pyruvate concentration, at pH 6.5 and 7.5 (Simongini et al., 2023). In addition, we found that the presence of 0.8 M trehalose in solutions containing rabbit LDH‐A counteracted the dissociation of the enzyme induced by acidic conditions, that is, pH 5 (Simongini et al., 2023).

Remarkably, the effects exerted by trehalose on individual steps of enzyme‐catalyzed reactions have not been investigated. Therefore, we considered it of interest to analyze by stopped‐flow assays the binding of *β*‐NADH to rabbit LDH‐A and the dissociation of the enzyme‐cofactor complex, as a function of trehalose concentration. In addition, to further inspect the action of trehalose on the catalytic performance of rabbit LDH‐A, we performed stopped‐flow and steady‐state assays in the presence of two different substrates, that is, pyruvate and oxaloacetate.

## 2 RESULTS

### 2.1 The binding of *β*‐NADH to rabbit muscle LDH

**FIGURE 1:**
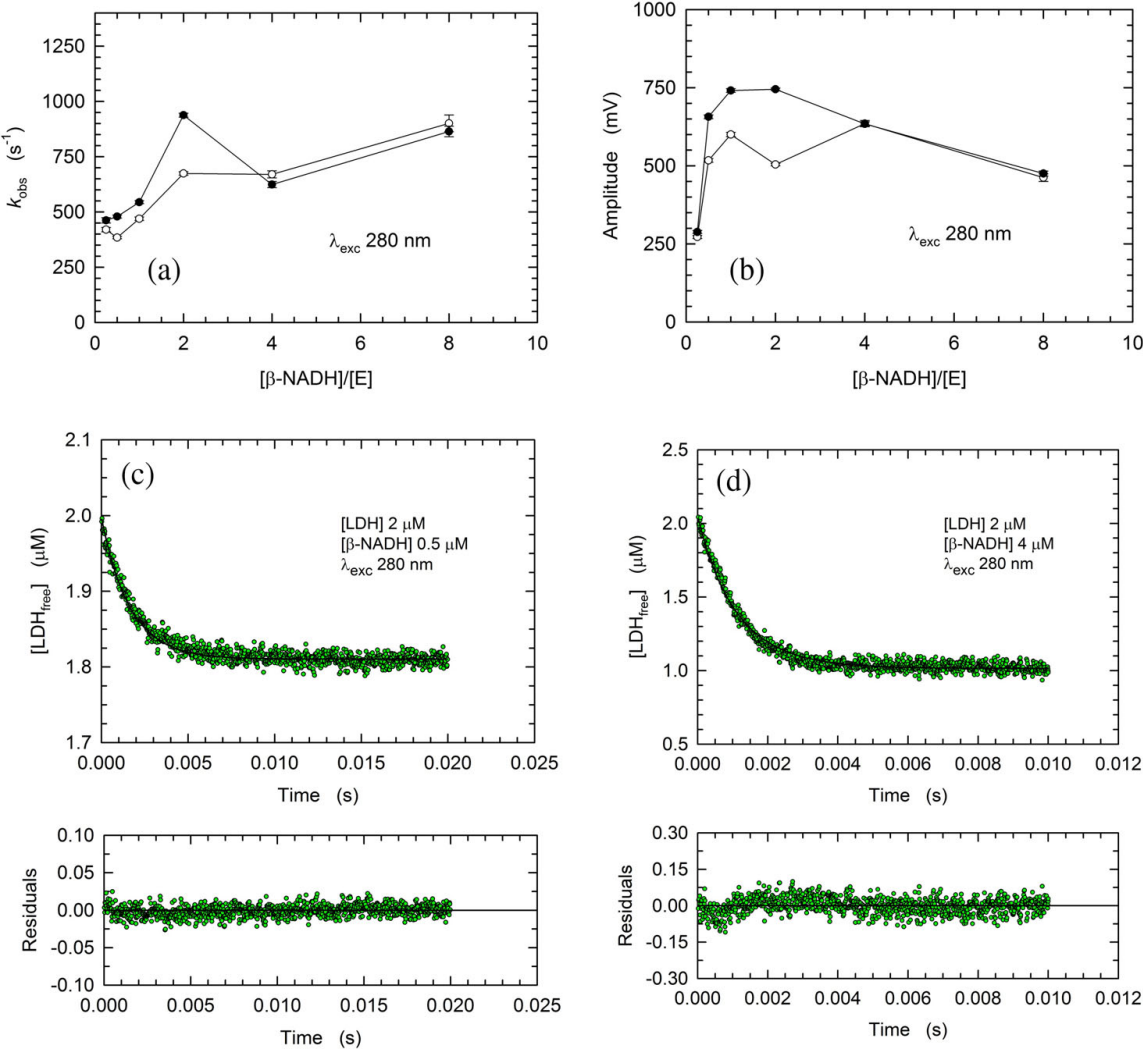
Rate constants, amplitudes, and kinetics of the fluorescence changes of rbLDH tryptophanes induced by mixing 4 μM enzyme with an equal volume of *β*‐NADH solutions containing different cofactor concentrations. (a), (b) Rate constants and amplitudes determined by fitting a single exponential equation to the experimental observations obtained in two sets of stopped‐flow assays (empty circles, Figure S1; filled circles, Figure S2). (c), (d) Best simulations (continuous lines) of the reversible binding of *β*‐NADH to rbLDH detected (green dots) by means of two stopped‐flow assays performed under the indicated conditions. The software COPASI was used to generate the best simulations. See Methods for details.

As a first test, we decided to inspect the binding of *β*‐NADH to rabbit LDH (rbLDH) by detecting the intramolecular Förster Resonance Energy Transfer (FRET) that occurs between the enzyme tryptophanes and the bound cofactor. To this aim, we performed stopped‐flow assays using a constant concentration of enzyme (2 μM of subunits) exposed to concentrations of cofactor corresponding to a *β*‐NADH/LDH molar ratio ranging from 0.25:1 to 8:1. In particular, by considering the *K*_D_ of the LDH‐cofactor complex as equal to 3.2–3.5 μM (Fromm, 1963; Stinson & Holbrook, 1973), the molar ratios considered translate into the association of only 1 or up to 4 *β*‐NADH molecules per tetrameric enzyme. Not surprisingly, the rate constant of the binding reaction was observed to increase as a function of cofactor concentration (Figures 1a, S1 and S2). In addition, the amplitude of the fluorescence changes triggered by the binding of *β*‐NADH featured a maximum at a *β*‐NADH/LDH molar ratio equal to 1 or 2 (Figure 1b). It should be noted that the decrease in *k*_obs_ and amplitude (Figure 1a,b) occurring at *β*‐NADH/LDH molar ratios higher than 2 is most likely due to the limited time resolution of the stopped‐flow technique. Nevertheless, using two assays performed at *β*‐NADH/LDH molar ratios equal to 0.25:1 and 2:1, we evaluated the second‐order rate constant (*k*_1_) of the reaction leading to the binding of cofactor by rbLDH. In particular, according to simulations carried out with the program COPASI (see Methods) we estimated values of *k*_1_ equal to (1.17 ± 0.01) × 10^8^ and (1.19 ± 0.11) × 10^8^ M^−1^ s^−1^ under conditions of 0.25:1 and 2:1 *β*‐NADH/LDH molar ratios, respectively (Figure 1c,d). These values are in reasonable agreement with that previously reported for rbLDH, equal to (1.82 ± 0.12) × 10^7^ M^−1^ s^−1^ (Greaney & Somero, 1980) and with those determined for pig heart and *Bacillus stearothermophilus* LDH, respectively equal to 9.5 × 10^7^ and 2.5 × 10^8^ M^−1^ s^−1^ (Deng et al., 2001; Nie et al., 2016). Moreover, the same simulations generated values for *k*_−1_ equal to 347 ± 3 s^−1^, and 344 ± 34 s^−1^ for the reaction observed in the presence of 0.5 and 4 μM *β*‐NADH, respectively (Figure 1c,d). Again, these values are in line with those previously reported for the corresponding rate constant featured by pig heart and *B. stearothermophilus* LDH, that is, 416 and 330 s^−1^, respectively (Deng et al., 2001; Nie et al., 2016).

### 2.2 Trehalose and the binding of *β*‐NADH to rabbit LDH

Taking into account how the concentration of *β*‐NADH affects its binding by rbLDH (Figure 1), we considered it of interest to test the effect of trehalose on the rate of generation of the enzyme‐cofactor complex in the presence of a *β*‐NADH/LDH molar ratio equal to 0.25:1 or 2:1.

**FIGURE 2:**
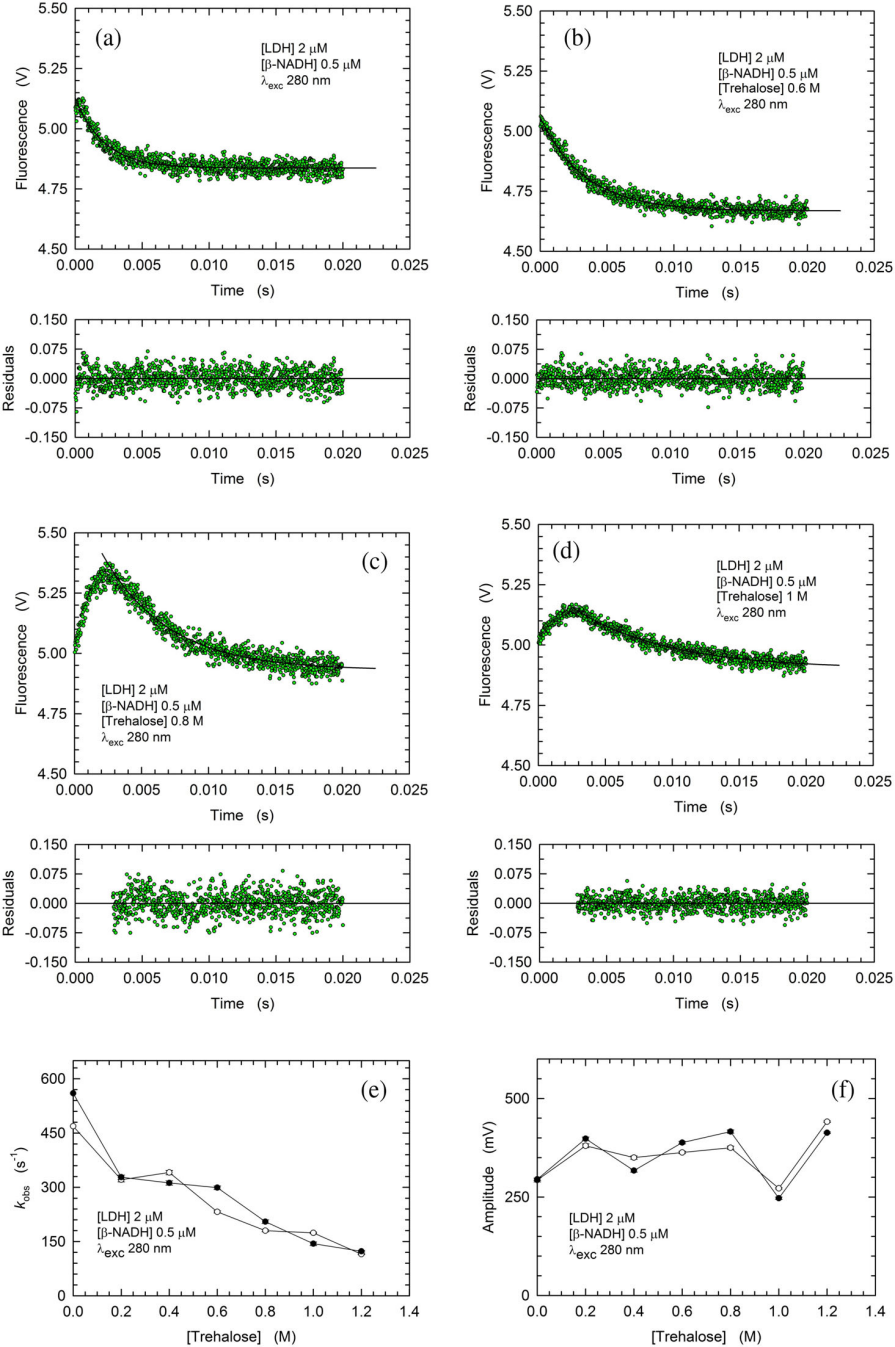
Kinetics, rate constants, and amplitudes of the fluorescence changes of rbLDH tryptophanes induced by mixing 4 μM enzyme with 1 μM *β*‐NADH. (a), (d) Kinetics of the fluorescence quenching of rbLDH tryptohanes triggered by *β*‐NADH binding in the absence (a) or in the presence of 0.6, 0.8, or 1 M trehalose (b, c, and d, respectively). (e), (f) Rate constants (e) and amplitudes (f) determined by fitting a single exponential equation to the experimental observations obtained in two sets of stopped‐flow assays (empty circles, Figures S3 and S4; filled circles, Figures S5 and S6).

First, we assayed the binding of *β*‐NADH under conditions enabling its association to only one subunit of tetrameric rbLDH, in the absence or in the presence of up to 1.2 M trehalose. Not surprisingly, the addition of trehalose to the reaction mixtures was found to decrease the rate according to which the enzyme binds *β*‐NADH. In particular, when trehalose was added at concentrations ranging from 0.2 to 0.6 M, the *k*_obs_ for the binding reaction did progressively decrease, down to approximately half the value determined in the absence of the disaccharide (Figure 2a,b,e, and S3–S6). This decrease of *k*_obs_ was not mirrored by major changes in the amplitude of the fluorescence changes triggered by *β*‐NADH binding (Figure 2f). When 0.8 M trehalose was added to reaction mixtures, we unexpectedly observed peculiar kinetics. Under this condition, we indeed detected an initial increase of fluorescence, followed by an exponential decay occurring 2 ms after mixing rbLDH and *β*‐NADH (Figures 2c, S4 and S6). Remarkably, similar kinetics, albeit characterized by a lower amplitude, occurred in the presence of 1 M trehalose (Figures 2d, S4 and S6). We interpret these observations as diagnostic of an initial closed‐to‐open conformational rearrangement of the enzyme, followed by the association with *β*‐NADH. Accordingly, the transition from a closed to an open form corresponds to an increased emission by the enzyme tryptophanes, whose fluorescence is subjected to a subsequent quenching concomitant with the binding of the cofactor. Moreover, we propose that in the presence of trehalose concentrations lower than 0.8 M, the enzyme could engage its closed‐to‐open conformational transition at rates fast enough to hamper the detection of this event. Curiously enough, in the presence of 1.2 M trehalose, we did not detect the initial increase of fluorescence occurring before the binding of *β*‐NADH by rbLDH (Figures S4C and S6C). This observation suggests that the addition of trehalose at 1.2 M limits the extent of conformational opening of the enzyme, and, in turn, that the cofactor is bound according to a different mode compared to that featured by rbLDH in the presence of lower concentrations of the disaccharide.

**FIGURE 3:**
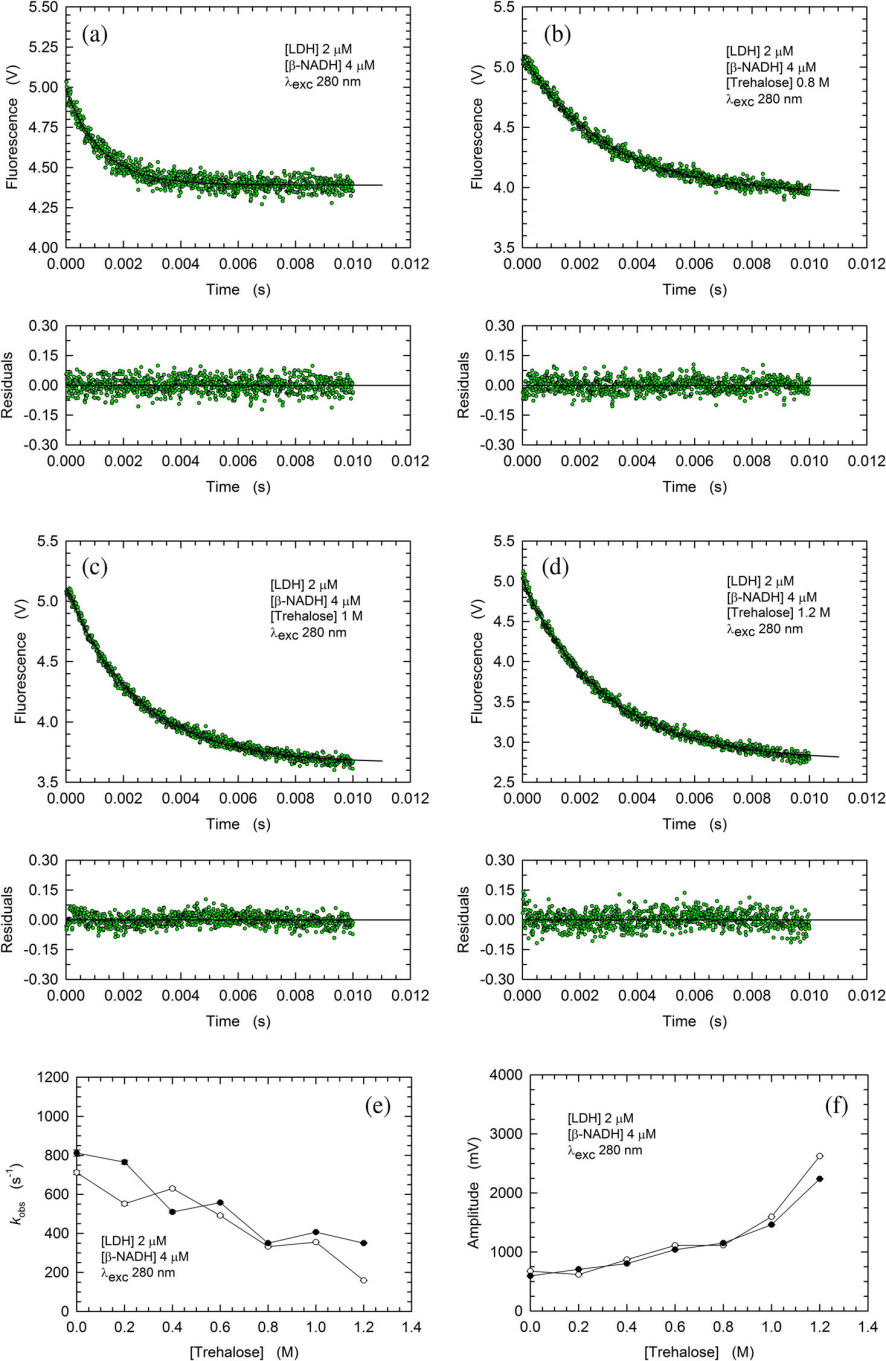
Kinetics, rate constants, and amplitudes of the fluorescence changes of rbLDH tryptophanes induced by mixing 4 μM enzyme with 8 μM *β*‐NADH. (a), (d) Kinetics of the fluorescence quenching of rbLDH tryptohanes triggered by *β*‐NADH binding in the absence (a) or in the presence of 0.8, 1, or 1.2 M trehalose (b, c, and d, respectively). (e), (f) Rate constants (e) and amplitudes (f) determined by fitting a single exponential equation to the experimental observations obtained in two sets of stopped‐flow assays (empty circles, Figures S7 and S8; filled circles, Figures S9 and S10).

Next, we assayed the association of *β*‐NADH to rbLDH under conditions of cofactor/enzyme molar ratio equal to 2:1, and in the absence or in the presence of trehalose at concentrations up to 1.2 M. Overall, the observed rates for the binding reaction and the corresponding amplitudes of the fluorescence changes were higher (Figure 3e,f) than those detected with the assays carried out using a *β*‐NADH/rbLDH molar ratio equal to 0.25:1 (Figure 2e,f). Nevertheless, the addition of trehalose to the reaction mixtures was found to negatively affect the rate of *β*‐NADH binding, according to a dependence on trehalose concentration similar to that determined with a molar excess of enzyme over cofactor (cf. Figures 2 and 3). On the contrary, the dependence on trehalose concentration of the amplitudes of the fluorescence changes triggered by *β*‐NADH binding was strongly affected by the cofactor/enzyme molar ratio. Indeed, a prominent increase in the observed amplitudes was only detected when the concentration of *β*‐NADH exceeded that of rbLDH, with mean values equal to 636 and 2433 mV determined in the absence and in the presence of 1.2 M trehalose, respectively (Figures 3f, and S7–S10). Moreover, it is important to note that the increase in trehalose concentration from 1 to 1.2 M triggered a sharp increase in the observed amplitudes, whose corresponding mean values were equal to 1530 and 2433 mV, respectively (Figures 3f, S8 and S10). This effect suggests that the presence of trehalose at concentrations higher than 1 M induces a marked compaction of the enzyme conformation, the occurrence of which translates into shortened distances between the enzyme tryptophanes and *β*‐NADH. Ultimately, these shortened distances should correspond to a magnification of the intramolecular FRET related to the binding of *β*‐NADH by rbLDH.

### 2.3 Dissociation of the rabbit LDH•*β*‐NADH complex

**FIGURE 4:**
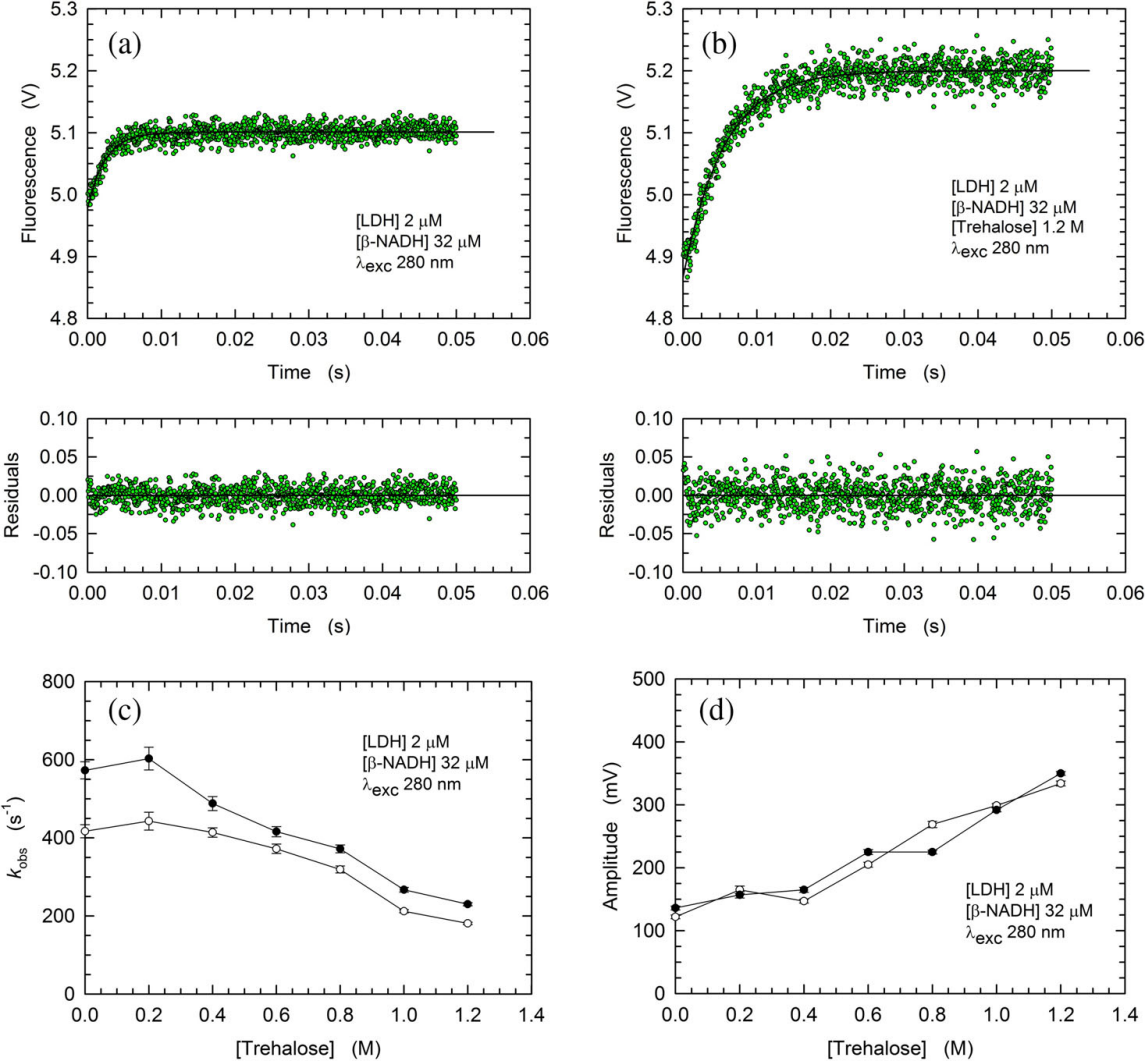
Kinetics, rate constants, and amplitudes of the fluorescence changes of rbLDH tryptophanes induced by mixing a solution containing 4 μM enzyme and 64 μM *β*‐NADH with an equal volume of buffer. (a), (b) Kinetics of the fluorescence increase of rbLDH tryptohanes triggered by the dilution‐induced dissociation of *β*‐NADH from the enzyme‐cofactor complex in the absence (a) or in the presence (B) of 1.2 M trehalose. (c), (d) Rate constants (c) and amplitudes (d) determined by fitting a single exponential equation to the experimental observations obtained in two sets of stopped‐flow assays (empty circles, Figures S11 and S12; filled circles, Figures S13 and S14).

To further test the effects of trehalose on the rbLDH/*β*‐NADH couple, we analyzed the dissociation of this binary complex in the absence and in the presence of the disaccharide (Figure 4). To this aim, stopped‐flow assays were performed by diluting a solution containing the enzyme‐cofactor complex with buffer only, thus inducing dissociation. In particular, for these assays we used a large cofactor/enzyme molar ratio (16:1), that is, a condition containing to a minimum the concentration of free enzyme before the dissociation reaction is triggered by dilution. Indeed, the *K*_D_ value equal to 3.2 μM previously reported for the rbLDH•*β*‐NADH complex (Fromm, 1963) translates the content of the enzyme syringe (4 μM enzyme and 64 μM *β*‐NADH) as composed of 95% of enzyme‐cofactor complex and 5% only of free rbLDH.

In agreement to what was previously observed for the binding reaction, trehalose triggered a decrease in the dissociation rate of the rbLDH/*β‐*NADH complex, and an increase in the amplitudes of the corresponding fluorescence changes (Figures 4c,d, and S11–S14). Approximately, the dissociation rate was halved and the observed amplitudes increased three‐fold over the interval of trehalose concentrations considered (Figures 4c,d, and S11–S14).

### 2.4 Binding of oxamate by the rabbit LDH•*β*‐NADH complex

**FIGURE 5:**
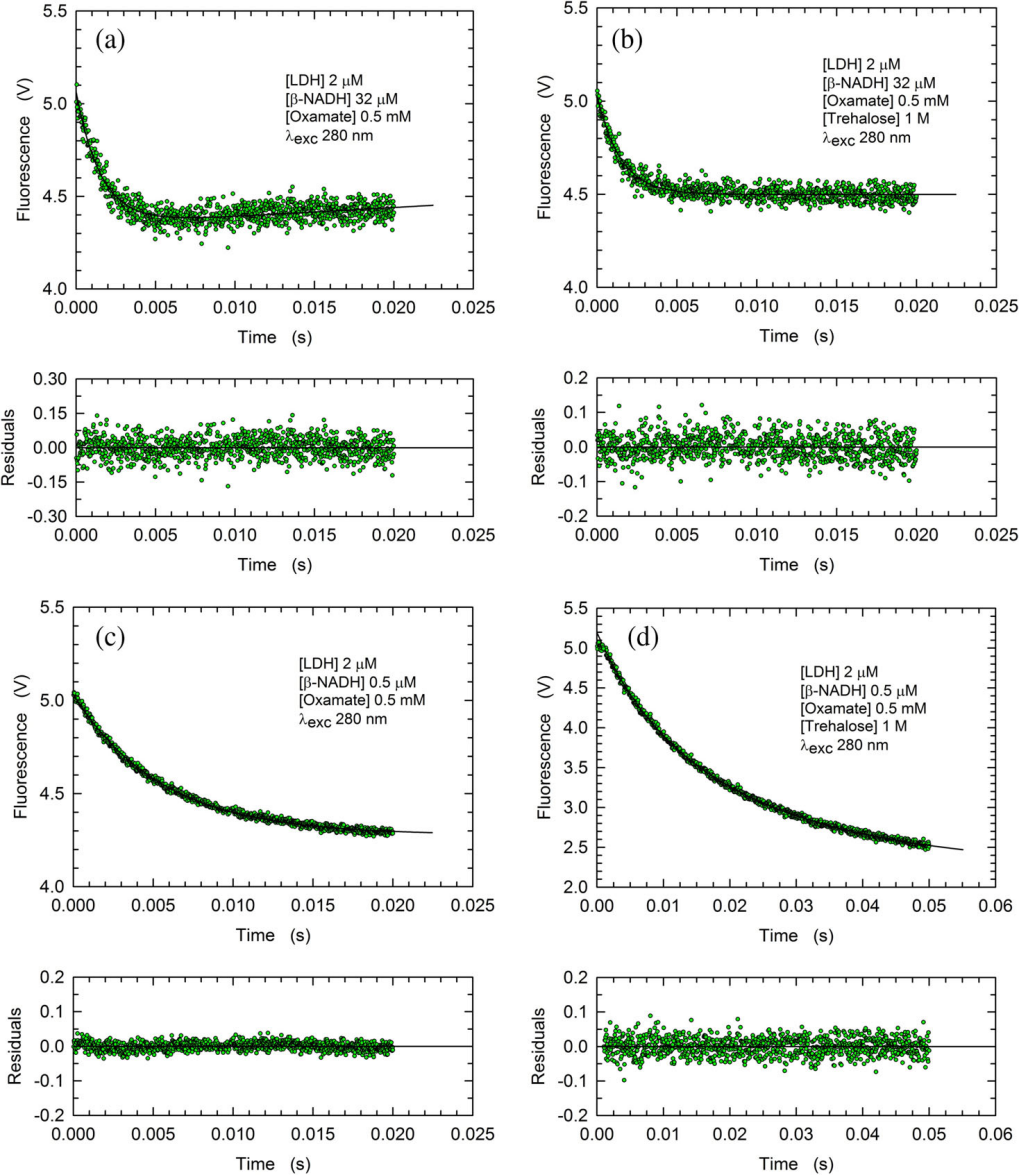
Kinetics of the fluorescence changes of rbLDH tryptophanes induced by mixing a solution containing 4 μM enzyme and *β*‐NADH with 1 mM oxamate. (a), (b) Kinetics of the fluorescence quenching of rbLDH tryptohanes triggered by mixing a solution containing 4 μM rbLDH and 64 μM *β*‐NADH with 1 mM oxamate in the absence (a) or in the presence (b) of 1 M trehalose. The continuous lines represent the best fit of a single exponential equation to the experimental observations. The fit in (a) was performed adding a linear component to the single exponential equation. (c), (d) Kinetics of the fluorescence quenching of rbLDH tryptohanes triggered by mixing a solution containing 4 μM rbLDH and 1 μM *β*‐NADH with 1 mM oxamate in the absence (c) or in the presence (d) of 1 M trehalose. The continuous lines represent the best fit of a single (c) or double (d) exponential equation to the experimental observations.

Quite a number of previous studies reported that the binding of oxamate, a competitive inhibitor structurally related to pyruvate, induces a conformational rearrangement of LDHs leading to the shielding of their active site from solvent (Nie et al., 2016; Waldman et al., 1988). This is accomplished by a movement of an enzyme loop, the displacement of which determines the closure of the active site and a substantial decrease of the fluorescence of the enzyme tryptophanes (Nie et al., 2016). Therefore, to analyze the kinetics of oxamate binding by rbLDH, we performed stopped‐flow assays observing the emission of tryptophanes, in the absence or in the presence of trehalose. As a first test, we exposed to 0.5 mM oxamate a solution containing 2 μM rbLDH and 32 μM *β*‐NADH, a molar ratio which translates into one cofactor molecule bound per subunit (see Methods). Rather surprisingly, under these conditions, both the *k*_obs_ and the signal amplitude were not greatly affected by the presence of 1 M trehalose (Figures 5a,b, S15A,B and Table 1). This similarity did not hold when 0.5 mM oxamate was mixed with a solution containing 2 μM rbLDH bound to at most one molecule of *β*‐NADH per tetrameric enzyme (Figures 5c,d, S15C,D, and Table 1). Indeed, only in the presence of trehalose, the observed exponential decay of fluorescence is composed of a fast and a slow phase (Table 1). In particular, the fast component is strictly similar to the single phase occurring when tetrameric rbLDH fully bound to *β*‐NADH is exposed to oxamate in the absence of trehalose (Table 1), and we propose that this event is related to the closure of the enzyme active site. In addition, we suggest that the slow component of the biphasic kinetics (Table 1) is diagnostic of a conformational rearrangement of the four subunits of rbLDH, triggered by the binding of oxamate.

**TABLE 1:** Rate constants and amplitudes of the fluorescence changes of rbLDH tryptophanes induced by mixing a solution containing 4 μM enzyme and *β*‐NADH with 1 mM oxamate.

[*β*‐NADH]/[LDH]	Assay time (ms)	Control		Trehalose (1 M)	
		*k*_obs_ (s^−1^)	Amplitude (mV)	*k*_obs_ (s^−1^)	Amplitude (mV)
16:1	20	642 ± 19	720 ± 10	680 ± 16	530 ± 8
16:1	10	683 ± 27	691 ± 12	617 ± 14	516 ± 6
1:4	20	183 ± 1	755 ± 2	133 ± 18	746 ± 171
1:4	20	‐	‐	41 ± 3	2209 ± 146
1:4	50	177 ± 1	758 ± 2	‐	‐
1:4	100	‐	‐	133 ± 3	1000 ± 30
1:4	100	‐	‐	40 ± 1	1976 ± 29

*Note*: Stopped flow assays were performed in the absence or in the presence of 1 M trehalose, using in the enzyme syringe a *β*‐NADH/LDH molar ratio equal to 16:1 or 1:4. The substrate syringe contained 1 mM oxamate. All the assays were carried out at 20°C in 50 mM Tris–HCl buffer, pH 7.5.

### 2.5 Dynamic light scattering (DLS) experiments

**FIGURE 6:**
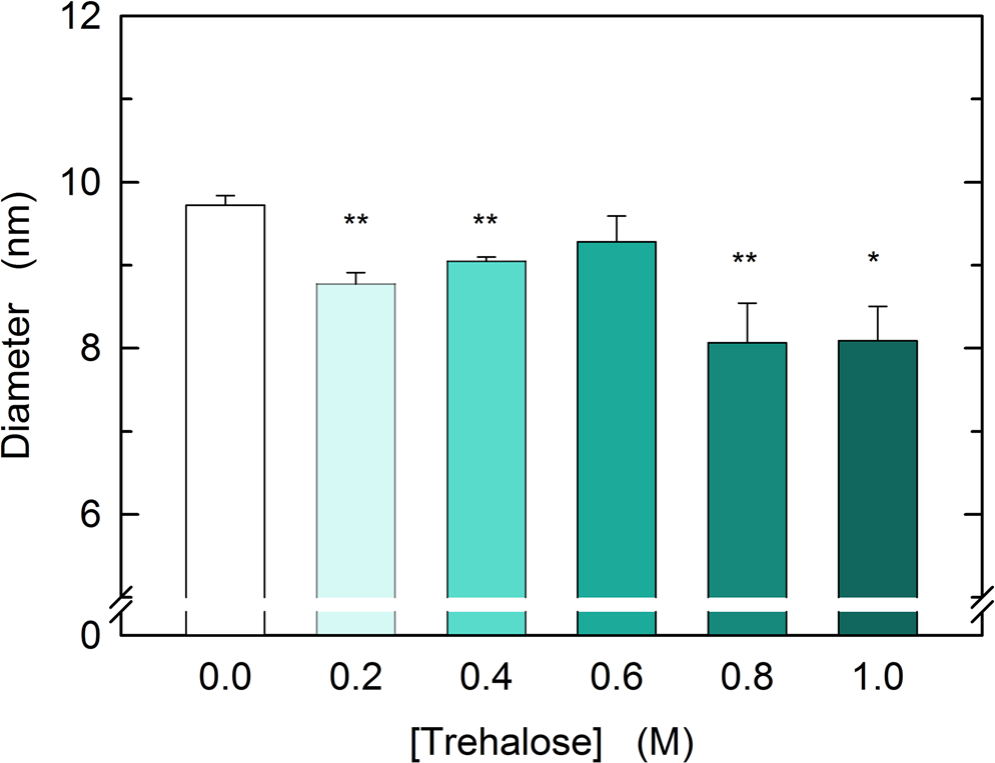
Effect of trehalose on the size of rabbit muscle lactate dehydrogenase. The diameter of rabbit muscle lactate dehydrogenase was determined by dynamic light scattering assays performed with solutions containing 3.8 μM enzyme (concentration of subunits) and poised at 20°C, in the absence or in the presence of trehalose at the indicated concentrations. All the analyzed samples were buffered using 50 mM Tris–HCl, pH 7.5. The error bars represent standard deviation (*n* = 3). Experimental mean values were compared by Student's *t*‐test (**, and * indicate *p* <0.001, and *p* <0.01, respectively).

We previously reported that the addition of 0.8 M trehalose to a solution buffered at pH 7.5 and containing rbLDH induces a compaction of the enzyme, the diameter of which was shortened by 0.7 nm (Simongini et al., 2023). Therefore, we considered it of interest to investigate how the size of rbLDH is affected by different concentrations of the disaccharide. Rather surprisingly, performing DLS assays, we observed that 0.2 M trehalose did suffice to decrease the diameter of rbLDH from 9.73 ± 0.11 to 8.78 ± 0.14 nm (Figures 6 and S16). Curiously enough, when the trehalose concentration was increased from 0.2 to 0.4 or 0.6 M, the compaction of the enzyme was found less pronounced with respect to that detected in the presence of 0.2 M disaccharide, with diameters equal to 9.05 ± 0.05 and 9.29 ± 0.31 nm, respectively (Figures 6 and S16). Furthermore, the strongest compaction of rbLDH was found at 0.8 and 1.0 M trehalose, in the presence of which we determined the diameter of the enzyme as equal to 8.07 ± 0.48 and 8.09 ± 0.41 nm, respectively (Figures 6, and S16).

### 2.6 Trehalose and the reduction of pyruvate by rabbit LDH

**FIGURE 7:**
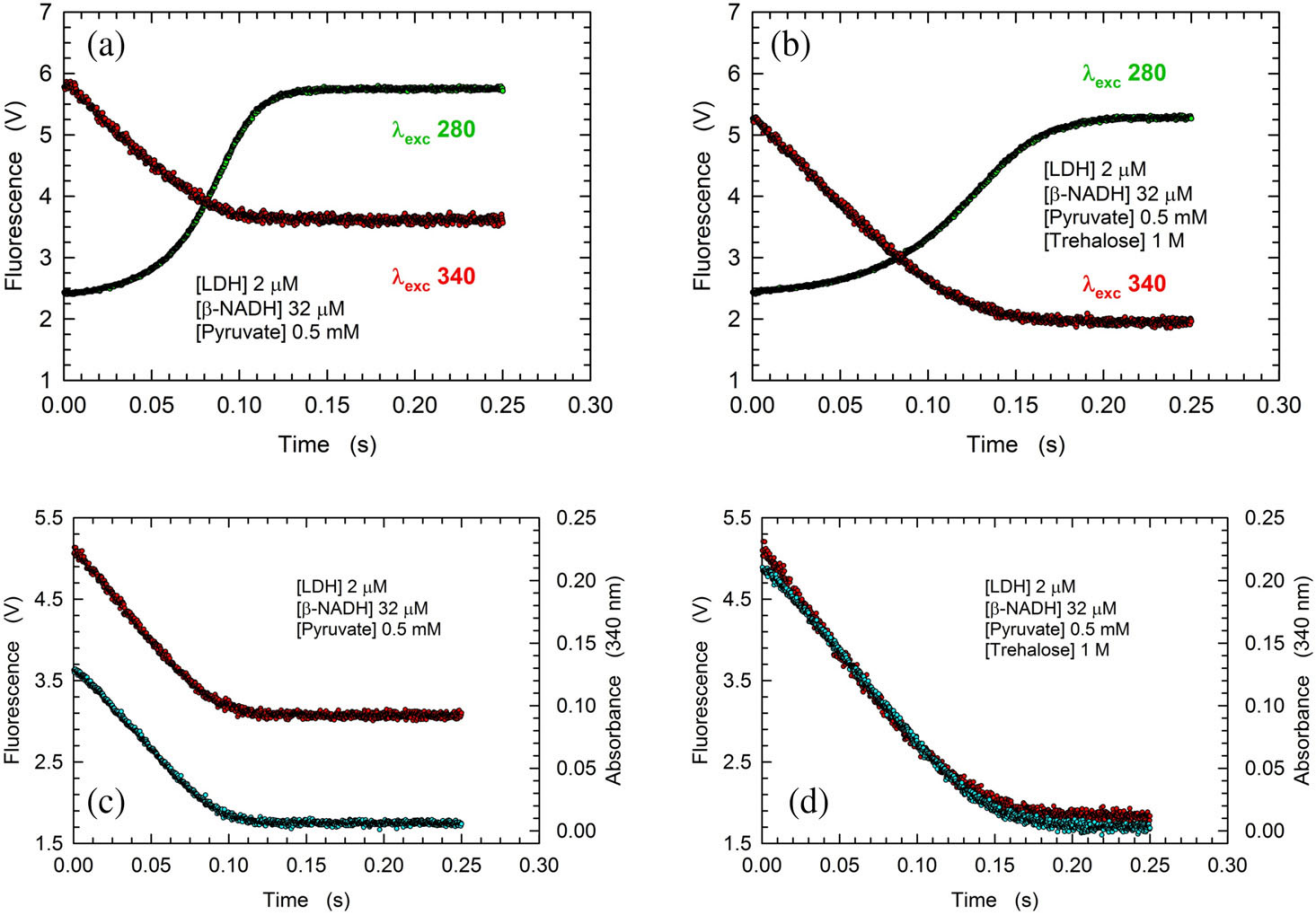
Trehalose action on the reduction of pyruvate catalyzed by rbLDH. (a), (b) Fluorescence changes of rbLDH tryptophanes (excited at 280 nm, green circles) and of *β*‐NADH (excited at 340 nm, red circles) triggered by mixing a solution containing 4 μM enzyme and 64 μM cofactor with 1 mM pyruvate as detected in the absence (a) or in the presence (b) of 1 M trehalose. (c), (d) Absorbance (at 340 nm, cyan circles) and fluorescence (red circles) changes related to the oxidation of *β*‐NADH catalyzed by rbLDH at the expense of pyruvate reduction in the absence (c) or in the presence (d) of 1 M trehalose.

Previous experiments performed with rbLDH showed that the reduction of pyruvate catalyzed by this enzyme is significantly affected by trehalose. In particular, it was reported that the values of *K*_m_ and *V*_max_ (*k*_cat_) of rbLDH both decreased when the catalytic action of the enzyme was assayed under steady‐state conditions in the presence of the disaccharide (Hernández‐Meza & Sampedro, 2018; Simongini et al., 2023). To further investigate this point, we performed stopped‐flow assays under conditions enabling a limited number of turnovers, in the absence or in the presence of 1 M trehalose (Figure 7). As expected, both the oxidation of *β*‐NADH and the concomitant increased emission by enzyme tryptophanes were slowed down by the addition of the disaccharide (Figure 7a,b). In particular, the reactions in the absence and in the presence of trehalose were observed to undergo completion within 150 and 250 ms, respectively (Figure 7a,b). This was confirmed by independent assays by which the oxidation of *β*‐NADH was determined by means of detecting the emission and the absorbance of the cofactor (Figure 7c,d).

### 2.7 Trehalose promotes the reduction of oxaloacetate by rabbit LDH

**FIGURE 8:**
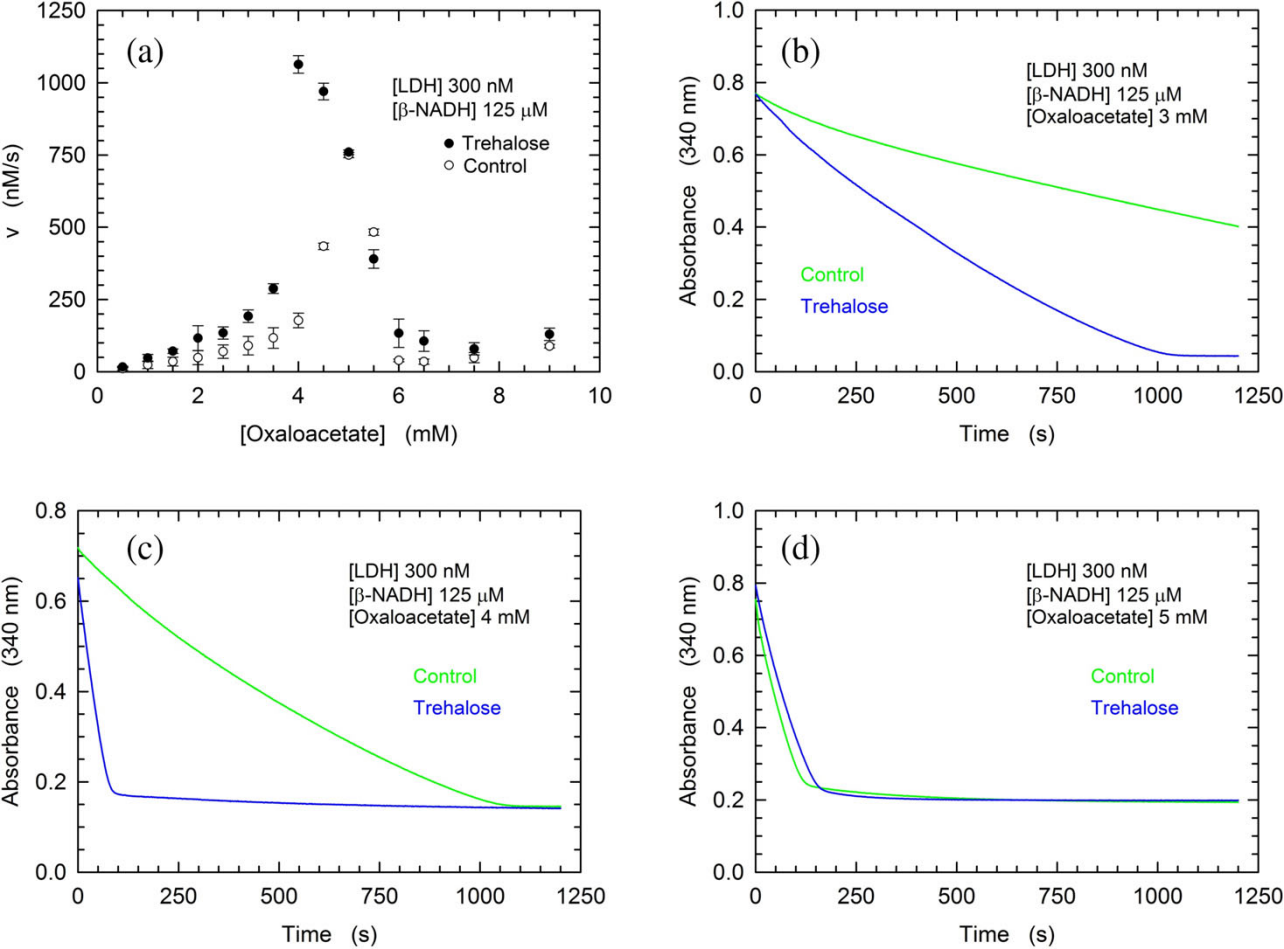
Kinetics of oxaloacetate reduction catalyzed by rbLDH in the absence or in the presence of 1 M trehalose. (a) Initial velocity of *β*‐NADH oxidation as a function of oxaloacetate concentration in reaction mixtures containing 300 nM rbLDH and 125 μM of the redox cofactor, in the absence (empty cicrles) or in the presence (filled circles) of 1 M trehalose. All the assays were performed at 20°C, pH 7.5 (50 mM Tris–HCl). (b), (d) Kinetics of *β*‐NADH oxidation catalyzed by rbLDH at the expense of 3, 4, or 5 mM oxaloacetate (b, c, and d, respectively) under the indicated conditions and in the absence (green lines) or in the presence (blue lines) of 1 M trehalose.

**FIGURE 9:**
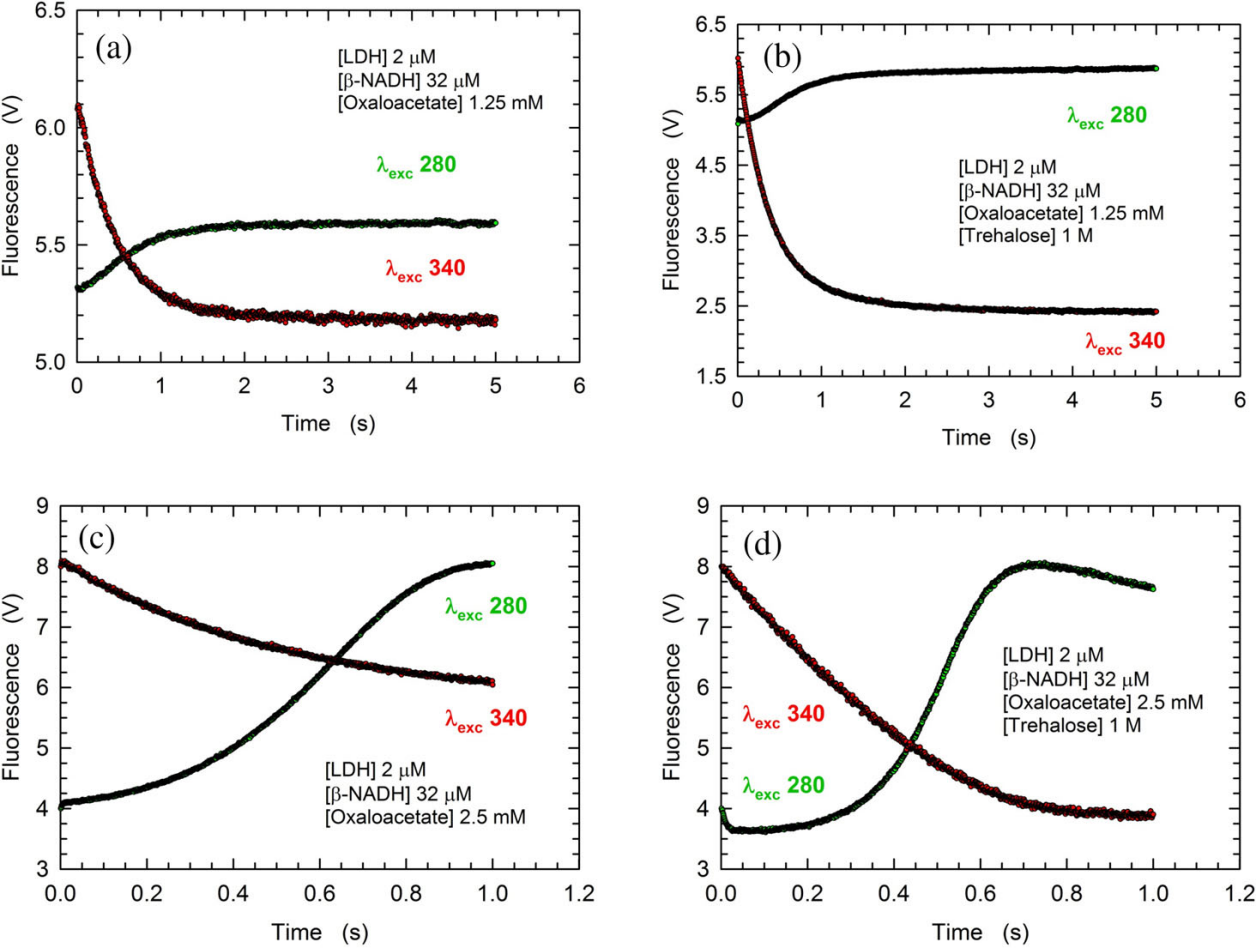
Trehalose effect on the reduction of oxaloacetate catalyzed by rbLDH. (a, b) Fluorescence changes of rbLDH tryptophanes (excited at 280 nm, green circles) and of *β*‐NADH (excited at 340 nm, red circles) triggered by mixing a solution containing 4 μM enzyme and 64 μM cofactor with 2.5 mM oxaloacetate as detected in the absence (a) or in the presence (b) of 1 M trehalose. (c), (d) Fluorescence changes of rbLDH tryptophanes (excited at 280 nm, green circles) and of *β*‐NADH (excited at 340 nm, red circles) triggered by mixing a solution containing 4 μM enzyme and 64 μM cofactor with 5 mM oxaloacetate as detected in the absence (c) or in the presence (d) of 1 M trehalose.

The effect triggered by trehalose on the reduction of pyruvate catalyzed by rbLDH was convincingly explained by the structural compaction and by the viscosity to which the enzyme is subjected when exposed to the disaccharide (Hernández‐Meza & Sampedro, 2018). In particular, the structural compaction and the high viscosity induced by trehalose are considered responsible for lowering the values of *K*_m_ and *V*_max_ featured by rbLDH. We were therefore intrigued to investigate the effects triggered by trehalose on the kinetic parameters of the enzyme acting on a substrate bulkier than pyruvate, for example, oxaloacetate. Rather surprisingly, by performing steady‐state assays, we observed that the reduction of oxaloacetate by rbLDH occurs at faster rates when 1 M trehalose is added to the assay mixture (Figure 8a). Quantitatively speaking, at 4 mM oxaloacetate, the reaction rate was found to be sixfold faster in the presence of the disaccharide (Figure 8a). Furthermore, it is interesting to note that trehalose enhanced the catalytic action of rbLDH on oxaloacetate at almost every substrate concentration tested, the only exception being the activities exerted by the enzyme at the expense of 5.0–5.5 mM oxaloacetate (Figure 8a–d). This exception is related to the higher sensitivity to substrate inhibition that rbLDH features in the presence of trehalose (Figure 8a–d). Notably, trehalose was also observed to enhance the rate of oxaloacetate reduction by means of stopped‐flow assays performed using 2 μM rbLDH and 32 μM *β*‐NADH, that is, under conditions limiting the extent of the reaction to 16 turnovers (Figure 9a–d).

## 3 DISCUSSION

We reported here on the effects triggered by trehalose on different reaction steps catalyzed by rbLDH and leading to the reduction of pyruvate or oxaloacetate at the expense of *β*‐NADH.

First, we assayed the rate of binding of the redox cofactor by rbLDH, in the absence of trehalose (Figure 1). In particular, under this condition, we estimated the *k*_1_ association constant as equal to (1.17 ± 0.01) × 10^8^ M^−1^ s^−1^, in good agreement with the values previously determined for *B. stearothermophilus* and pig heart LDH (Deng et al., 2001; Nie et al., 2016). It should be noted that the *k*_1_ of the bacterial enzyme was evaluated by means of the T‐jump technique, whose time resolution enabled the identification of two conformational rearrangements of LDH occurring before and after the binding of *β*‐NADH (Nie et al., 2016). Moreover, these events were recognized as the open‐to‐closed reversible transition of the active site loop in the free enzyme and in the cofactor‐rbLDH complex, respectively (Nie et al., 2016). It should also be noted that the binding of *β*‐NADH and of the *α*‐ketoacid substrate can only be exerted by the enzyme featuring the active site loop in the open conformation (Nie et al., 2016).

We then determined the rate of binding of *β*‐NADH by rbLDH, in the absence and in the presence of trehalose. When the binding of the cofactor was assayed under a *β*‐NADH/LDH molar ratio equal to 0.25:1, the observed association rate constant decreased as the concentration of the disaccharide was increased (Figure 2). More importantly, at 0.8 and 1 M trehalose, the fluorescence of rbLDH tryptophanes was found to undergo an initial increase, to which followed a decrease of similar amplitude (Figure 2c,d). We interpret these unprecedented observations as related to a primary transition, occurring in about 2 ms, poising the enzyme in the open conformation, followed by the binding of *β*‐NADH (Figure 2c,d). The binding of the redox cofactor was also analyzed at a *β*‐NADH/LDH molar ratio equal to 2:1. Not surprisingly, under this condition, the reaction rates were faster (Figure 3) than those detected in the presence of a lower concentration of *β*‐NADH (Figure 2). Moreover, trehalose was found to decrease the rate of cofactor binding and to increase the amplitude of the associated fluorescence changes, with the magnitude of these effects being proportional to the concentration of the disaccharide (Figure 3). Furthermore, stopped‐flow assays of the dissociation of the rbLDH‐cofactor complex revealed similar effects triggered by trehalose (cf. Figures 3 and 4). Overall, our observations on the association of *β*‐NADH to rbLDH and those on its dissociation from the enzyme‐cofactor complex suggest that trehalose slows down the structural fluctuations of rbLDH and the motions of the active site loop. This translates into a slower transition of the enzyme from the closed to the open conformation, which, in turn, implies a slower binding/dissociation of *β*‐NADH. Furthermore, these events affected by trehalose are coupled with an increase in the magnitude of the intramolecular FRET occurring from the enzyme tryptophanes to *β*‐NADH (Figures 3 and 4). We propose that this increase is related to the compaction of the rbLDH structure induced by trehalose (Figure 6), which is mirrored by the shortening of the tryptophan‐cofactor distances. It should indeed be noted that the efficiency of FRET does inversely depend on the sixth power of the distance between the donor (tryptophan) and the acceptor (*β*‐NADH) partners (Szabó et al., 2022).

Next, we tested the effect exerted by trehalose on the binding of oxamate to rbLDH. Accordingly, binding assays were performed in the absence or in the presence of the disaccharide, using 2 μM rbLDH and 0.5 mM oxamate, that is, a concentration of this competitive inhibitor exceeding the value of its *K*_i_, which was reported as equal to 0.23 mM (Tuengler et al., 1980). Therefore, under these conditions, the majority (70%) of rbLDH subunits can bind oxamate. However, we constrained the binding of oxamate by exposing it to rbLDH fully bound to *β*‐NADH or an enzyme‐cofactor complex containing at most one subunit associated with *β*‐NADH (Figure 5). Rather surprisingly, the rate of oxamate binding was not affected by the presence of 1 M trehalose when rbLDH was saturated with cofactor (Figures 5a,b, S15A,B, and Table 1). Moreover, the association of oxamate to rbLDH containing no more than one molecule of *β*‐NADH per enzyme tetramer was only partially affected by trehalose, the presence of which induced a slow conformational rearrangement occurring after the binding event (Figures 5c,d, S15C,D, and Table 1).

Among the factors acting on reaction rates, viscosity represents a determinant of primary importance. Concerning rabbit LDH, it was reported that different viscogens induce a decrease of the enzyme *K*_m_ and *V*_max_ when the catalytic action on pyruvate is considered (Demchenko et al., 1989; Hernández‐Meza & Sampedro, 2018; Simongini et al., 2023). In particular, the presence of 0.8 M trehalose was found to halve the values of both kinetic parameters (Hernández‐Meza & Sampedro, 2018; Simongini et al., 2023), an effect quantitatively similar to that triggered by 2.8 M glycerol (Demchenko et al., 1989). Here we show that the velocity of the reduction of pyruvate by rbLDH is negatively affected in the presence of 1 M trehalose (Figure 7). By considering how trehalose acts on different steps of this reaction (Figures 2, 3, 4, 5), we propose that the inhibiting action of trehalose represents the output of slowing down the binding of *β*‐NADH to rbLDH. Moreover, taking into account the ordered mechanism to which rbLDH obeys, this action of trehalose should hold when the enzyme catalyzes the reduction of oxaloacetate. Accordingly, our unprecedented observation that the activity of rbLDH at the expense of oxaloacetate is exerted at higher rates in the presence of 1 M trehalose is rather intriguing (Figure 8). Furthermore, when the enzyme was exposed to this dicarboxylic substrate we observed peculiar kinetics. Overall, the dependence of the enzyme activity on the concentration of oxaloacetate (Figure 8a) indicates the following: (i) the reaction initial velocity does linearly depend on substrate concentration up to 2.5 and 3.5 mM, in the presence and in the absence of trehalose, respectively; in particular, under these conditions the catalytic action of rbLDH in the presence of trehalose is twice that observed in the absence of the disaccharide (Figure S17); moreover, by supplementing with trehalose a reaction mixture containing 1.25 mM oxaloacetate the amplitude of the fluorescence changes of enzyme tryptophanes is increased about twofold (Figure 9a,b); (ii) at oxaloacetate concentrations higher than 2.5–3.5 mM, the enzyme activity increases exponentially, implying the binding of substrate to an accessory site featuring activating competence (Figure 8a); (iii) at 4 mM oxaloacetate the reaction velocity in the presence of trehalose is sixfold higher than in its absence; (iv) at oxaloacetate concentrations higher than 4–5 mM substrate inhibition was clearly detected (Figure 8a). Concerning the enhancement of rbLDH activity by oxaloacetate (Figure 8a), we propose that the activating locus corresponds to the allosteric site previously found to trigger non‐competitive inhibition of the rbLDH catalytic action on pyruvate (Alam et al., 2017). Besides substrate inhibition, the decrease of rbLDH activity detected at oxaloacetate concentrations higher than 4–5 mM (Figure 8a) could be partially due to the dissociation of the tetrameric enzyme induced by the ketoacid. To test this, we performed ultrafiltration assays using tetrameric rbLDH exposed or not to oxaloacetate (see Methods). By this means we detected a very slight, if at all, dissociation when the enzyme was incubated with 4 mM oxaloacetate. Indeed, we recovered in the retentate 98 ± 3 and 97% ± 2% of the total enzyme subjected to ultrafiltration in the absence and in the presence of 4 mM oxaloacetate, respectively. Interestingly enough, when 6 mM oxaloacetate was added to rbLDH solutions we recovered in the retentate 85% ± 3% only of the total enzyme loaded. Accordingly, the observed decrease of rbLDH activity occurring at high oxaloacetate concentrations (Figure 8a) is due to both substrate inhibition and dissociation of the tetrameric enzyme.

Notably, it was reported that the oxygen evolution activity of spinach photosystem II (PSII) core complexes is significantly stimulated by 1 M trehalose (Mamedov et al., 2015; Mamedov et al., 2018). In particular, the rate of oxygen evolution was observed to increase 2.5 times in the presence of the disaccharide (Mamedov et al., 2015), and it was shown that trehalose interacts with the donor side of PSII (Mamedov et al., 2018). Furthermore, it was proposed that trehalose acts on PSII by promoting a conformation of this photosynthetic complex favorable for the release of products (Mamedov et al., 2018). Accordingly, to further inspect the enhancement triggered by trehalose of the rbLDH activity on oxaloacetate, it will be interesting to analyze the effect of the disaccharide on the dissociation of malate and *β*‐NAD^+^ from rbLDH.

It is important to note that the catalytic action of rbLDH at the expense of oxaloacetate is much weaker when compared to the activity observed in the presence of pyruvate. In particular, here we report that in the absence of trehalose the highest initial velocity was determined as equal to 750 nM/s in the presence of 5 mM substrate and 300 nM enzyme (Figure 8a). When the catalytic action at the expense of pyruvate was assayed under the same conditions the *V*_max_ of the reaction was estimated as equal to 2.1 μM/s in the presence of 12.4 nM enzyme (Simongini et al., 2023). Accordingly, the activity exerted in the presence of the monocarboxylic acid is approximately 70‐fold higher than that observed when oxaloacetate is the substrate. This is in agreement with an analogous comparison reported for pig heart LDH, according to which the activity with pyruvate greatly outperforms that with oxaloacetate (Parker & Holbrook, 1981). To further ascertain the effect of trehalose on the catalytic action of LDHs at the expense of oxaloacetate, we assayed the activity of human muscle LDH. Remarkably, assays performed in the presence of oxaloacetate at 3 or 4 mM revealed that trehalose strongly enhances the activity of the human enzyme on this substrate (Figure S18), suggesting that the effect elicited by the disaccharide is relevant among mammalian LDHs.

**FIGURE 10:**
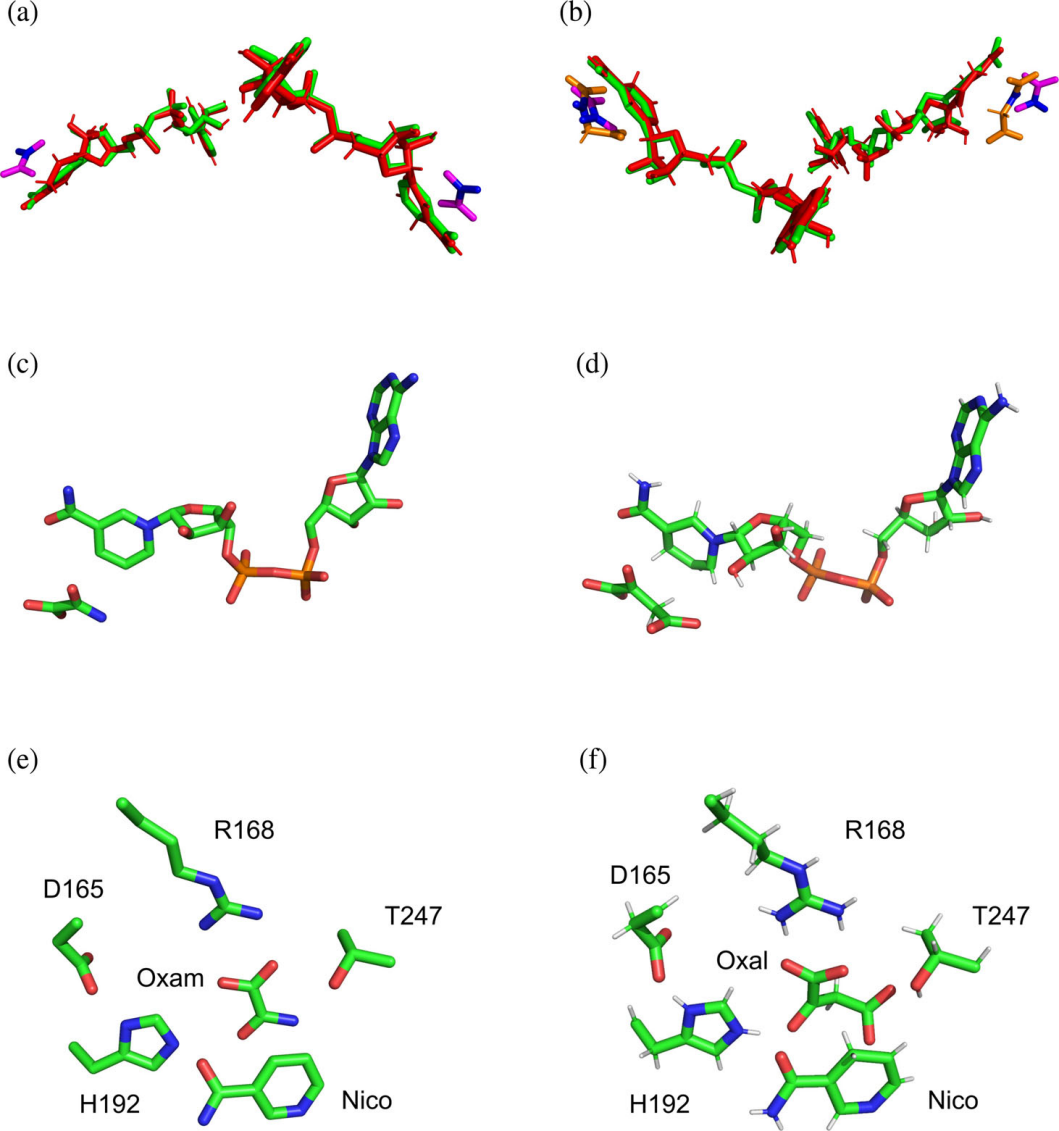
Structural details of the rbLDH active site containing *β*‐NADH and oxamate or oxaloacetate. (a) Conformation of *β*‐NADH bound to rbLDH chains A (left) and B (right) in the enzyme exposed to oxamate (green sticks, PDB file 3h3f) or oxaloacetate (red sticks, PDB file 5nqq). Oxamate is shown with magenta sticks, indicating the carbonyl moiety in blue. Oxaloacetate is not associated to these two enzyme chains. (b) Conformation of *β*‐NADH bound to rbLDH chains C (left) and D (right) in the enzyme exposed to oxamate (green sticks) or oxaloacetate (red sticks). Oxamate and oxaloacetate are shown with magenta and orange sticks, respectively, indicating the carbonyl moiety in blue. (c), (d) Structure of *β*‐NADH bound to rbLDH chain C in the enzyme exposed to oxamate (c) or oxaloacetate (d) substrate. (e), (f) Structure of the rbLDH active site containing oxamate (e) or oxaloacetate (f).

Rather surprisingly, the quaternary structure of rbLDH determined in the presence of *β*‐NADH and oxaloacetate reveals that each of the four subunits is associated with the redox cofactor, whereas only two subunits do also contain the ketoacid (Figure 10a,b) (Alam et al., 2017). Accordingly, we propose that trehalose induces a full occupancy of the rbLDH subunits by oxaloacetate, therefore inducing the observed doubling of reaction velocity at substrate concentrations up to 2.5 mM (Figures 8a and S17). As previously mentioned, at higher oxaloacetate concentrations, the activating action exerted by trehalose on rbLDH is even more pronounced (Figure 8). Interestingly enough, the structure of the ternary complex rbLDH‐NADH‐oxaloacetate features a distorted (puckered) conformation of the cofactor nicotinamide moiety, whereas in the presence of oxamate, the nicotinamide ring of *β*‐NADH features a planar conformation (Figure 10c,d). Quantitatively speaking, this distortion translates into elongated distances, induced by oxaloacetate, between the C4 and the N1 or the C7 of nicotinamide (Figure 10e,f, and Table 2). Furthermore, and more importantly, the distance from the nicotinamide C4 and the oxaloacetate carbon (C3) accepting the hydride from *β*‐NADH is also extended with respect to the distance to the corresponding carbon (C1) of oxamate (Figure 10e,f, and Table 2). In addition to this, it should be noted that H192 features a shorter distance from oxaloacetate than from oxamate, and that T247 interacts with the additional carboxylate of oxaloacetate (Figure 10e,f, and Table 2). Accordingly, we suggest that the trehalose‐induced compaction of rbLDH structure favors the reduction of oxaloacetate via a conformational rearrangement of *β*‐NADH and of the carboxylate proximal to T247. It should indeed be considered that the conformational transition leading to active site closure is the rate‐limiting step of the reaction catalyzed by LDHs at the expense of pyruvate, whereas in the presence of ketoacids bulkier than pyruvate, it is the hydride transfer step that becomes limiting (Wilks et al., 1990). Interesting and elegant experiments performed with pig heart LDH did indeed show that the hydride transfer rate can be increased more than twofold (at 25°C) by the addition of 1 M TMAO to the reaction mixtures (Zhadin & Callender, 2011). Moreover, it is important to note that when RNase‐containing solutions were supplemented with 1 M TMAO, a significant decrease of the enzyme Stokes radius was observed (Qu et al., 1998). Accordingly, the compaction of rbLDH triggered by 1 M trehalose (Figure 6) does quite likely favor the reduction of oxaloacetate by acting on the hydride transfer rate.

**TABLE 2:** Structural features of the rbLDH active site containing oxamate or oxaloacetate.

Atoms	Distance (Å) LDH‐Oxamate	Distance (Å) LDH‐oxaloacetate
C4‐N1 (Nicotinamide)	2.7	3.0
C4‐C7 (Nicotinamide)	2.5	2.6
C4(Nicotinamide): ‐C1(Oxam), ‐C3(Oxal)	3.3	3.5
H192(Nε2): ‐O1(Oxam), ‐O3(Oxal)	3.2	2.4
T247(Oγ): ‐O2(Oxam), ‐O1(Oxal)	3.0	2.8
T247(Cγ): ‐O2(Oxam), ‐O1(Oxal)	3.3	3.1

*Note*: The distances shown were determined with PyMol using the 3h3f and 5nqq PDB files (corresponding to the quaternary structures of the rbLDH/*β*‐NADH/oxamate and rbLDH/*β*‐NADH/oxaloacetate ternary complexes, respectively).

## 4 CONCLUSIONS

The unprecedented observation reported here that the addition of 1 M trehalose to reaction mixtures can enhance the catalytic action of rabbit muscle LDH is rather intriguing. Indeed, this enhancement occurs despite the inhibitory effect that the viscosity induced by the disaccharide exerts on reaction rates. It is therefore our hope that further analyses will contribute to a detailed understanding of the activating action of trehalose and, possibly, to its detection in enzymes other than rabbit LDH.

## 5 MATERIALS AND METHODS

### 5.1 Reagents

Buffers, enzyme substrates, and trehalose were purchased from Merck‐Millipore (St. Louis, MO, USA).

### 5.2 Rabbit LDH

Lactate dehydrogenase from rabbit skeletal muscle (LDH‐A, enzyme suspension in 3.2 M ammonium sulfate, lot 71511220) was obtained from Roche (Basel, Switzerland). LDH‐A was extensively dialyzed against 50 mM Tris–HCl (pH 7.5), and the dialyzed enzyme was concentrated to approximately 10 mg/mL with an Amicon ultrafiltration cell equipped with a YM100 membrane. Aliquots of the concentrated enzyme solution were supplemented with glycerol (20%, v/v) and stored at −20°C until used.

### 5.3 Human LDH

Lactate dehydrogenase from human muscle (LDH‐A) was overexpressed in *Escherichia coli* as previously described (Stefan et al., 2024). Proteins were extracted from cells subjected to overexpression, and the human LDH was purified by standard chromatographic techniques (Stefan et al., 2024). The best fractions (according to SDS‐PAGE analysis) eluted from the final purification step, that is, size exclusion chromatography, were pooled, concentrated, supplemented with glycerol (20%, v/v), and stored at – 20°C until used.

### 5.4 Activity assays

The enzyme‐catalyzed reduction of oxaloacetate was assayed by determining the decrease in absorbance at 340 nm related to the oxidation of *β*‐NADH. Reaction mixtures contained 50 mM Tris–HCl (pH 7.5), 125 μM *β*‐NADH, 300 nM enzyme, and variable concentrations of oxaloacetate. The extinction coefficient of *β*‐NADH at 340 nm was considered equal to 6.22 × 10^3^ M^−1^ cm^−1^ (Bernofsky & Wanda, 1982). All the assays were performed at least in duplicate at 20°C, using a Cary 300 Bio spectrophotometer and starting reactions by enzyme addition. Protein concentration was determined spectrophotometrically by recording the UV absorption spectra of the purified enzymes (wavelength interval: 250–400 nm) and using extinction coefficients at 280 nm equal to 43,690 and 45,170 M^−1^ cm^−1^ for the rabbit and human enzyme, respectively.

### 5.5 Stopped‐flow assays

A KinTek SF2004 stopped‐flow instrument (KinTek, Snow Shoe, PA, USA) was used to observe the time course of *β*‐NADH oxidation and the kinetics of fluorescence changes of the rabbit LDH‐A tryptophanes. The oxidation of *β*‐NADH and the fluorescence of LDH‐A tryptophanes were determined by exciting samples at 340 and 280 nm, respectively, and detecting emitted light using a longpass filter (cut‐on wavelength 350 nm). All the assays were performed in 50 mM Tris–HCl, pH 7.5 (T‐buffer). The enzyme syringe was filled with 4 μM rabbit LDH in T‐buffer, containing or not *β*‐NADH. The substrate syringe contained, in T‐buffer, pyruvate, oxamate, or oxaloacetate when the enzyme syringe contained *β*‐NADH, or was filled with *β*‐NADH in T‐buffer. It is important to note that considering the *K*_D_ of the enzyme‐cofactor complex equal to 3.2 μM (Fromm, 1963), the assays of oxamate binding performed in the presence of 2 μM rbLDH and 32 μM *β*‐NADH correspond to a condition under which essentially all subunits of the tetrameric enzyme are bound to the redox cofactor. Samples were equilibrated at 20°C before performing the assays. An observation cell of 0.5 cm path length was used. Unless otherwise stated, concentrations of enzyme and substrates do always refer to those obtained after mixing. For each measurement, 3–20 traces were averaged.

### 5.6 Simulation of the kinetics of *β*‐NADH binding to rbLDH

To evaluate the rate constants associated with the binding of *β*‐NADH to rbLDH and to the dissociation of the enzyme‐cofactor complex, the software COPASI was used (Hoops et al., 2006). In particular, the kinetics of the fluorescence changes observed by stopped‐flow assays after mixing 4 μM rbLDH with *β*‐NADH at 1 or 8 μM (Figure 1c,d, respectively) were interpreted as the output of the reversible association of *β*‐NADH to rabbit LDH. Accordingly, taking into account the value (3.2 μM) for the dissociation constant of the enzyme‐cofactor complex reported by Fromm (Fromm, 1963) the fluorescence values were converted into the concentration of free rbLDH. The best simulation of the observed kinetics was finally obtained using the evolutionary programming with 200 generations and a population size equal to 20.

### 5.7 Dissociation assays

To test the effect, if any, induced by oxaloacetate on the oligomeric state of rbLDH, ultrafiltration experiments were performed. Reaction mixtures containing (in 50 mM Tris–HCl, pH 7.5) 3 μM tetrameric enzyme, 125 μM *β*‐NADH, and 4 or 6 mM oxaloacetate were prepared and immediately loaded onto disposable ultrafiltration cells (Amicon Ultra, 100 kDa M_r_ cutoff, volume 0.5 mL). Control mixtures devoid of oxaloacetate were also prepared. The ultrafiltration cells were centrifuged for 15 s at 14,000 × *g*; the retentate and the eluate were collected and then subjected to the analysis of protein concentration (Bradford, 1976).

### 5.8 Dynamic light scattering

DLS experiments were performed with a Malvern Panalytical (Malvern, UK) Zetasizer Nano ZS system. All the measurements were recorded at 20°C using solutions previously filtered with 0.2 μm filters. Scattering was evaluated at an angle of 173°. The viscosity of the different solutions containing trehalose was considered according to values previously reported (Elias & Elias, 1999; Galmarini et al., 2011; Rampp et al., 2000; Sampedro et al., 2002). Raw data were analyzed with the Zetasizer software (Malvern Panalytical), release 7.11.

### 5.9 Structural analysis

The quaternary structures of rabbit muscle LDH bound to *β*‐NADH and oxamate or oxaloacetate (PDB files 3h3f and 5nqq, respectively) were inspected and represented using the PyMol software (DeLano, 2004).

## AUTHOR CONTRIBUTIONS

**Alessandra Stefan:** Investigation. **Alejandro Hochkoeppler:** Conceptualization; investigation; writing – review and editing; supervision; formal analysis; writing – original draft.

## CONFLICT OF INTEREST STATEMENT

The authors declare no conflict of interest.

## Supporting information


**Data S1:** Supplementary Information

## Data Availability

The data that support the findings of this study are available from the corresponding author upon reasonable request.
