# Supplementary material for: Not only an inhibitor: Trehalose enhances the catalytic action exerted on oxaloacetate by rabbit lactate dehydrogenase
Source: Protein Sci. 2025 Sep 13;34(10):e70304. doi: 10.1002/pro.70304 (PMC12432435; doi:10.1002/pro.70304)
Supplement: Supplementary file 1 — Data S1: Supplementary Information [file PRO-34-e70304-s001.pdf]

Supporting information for:

Not only an inhibitor: trehalose enhances the catalytic action exerted on oxaloacetate by rabbit lactate dehydrogenase

Alessandra Stefan[$£], Alejandro Hochkoeppler[$£*]

[$]Department of Pharmacy and Biotechnology, University of Bologna, Via Gobetti 87, 40129 Bologna (Italy)

[£]CSGI, University of Firenze, Via della Lastruccia 3, 50019 Sesto Fiorentino (Italy)

[*]Corresponding Author. e-mail: a.hochkoeppler@unibo.it

Table of contents:

Figures S1-S18 pS2-S19

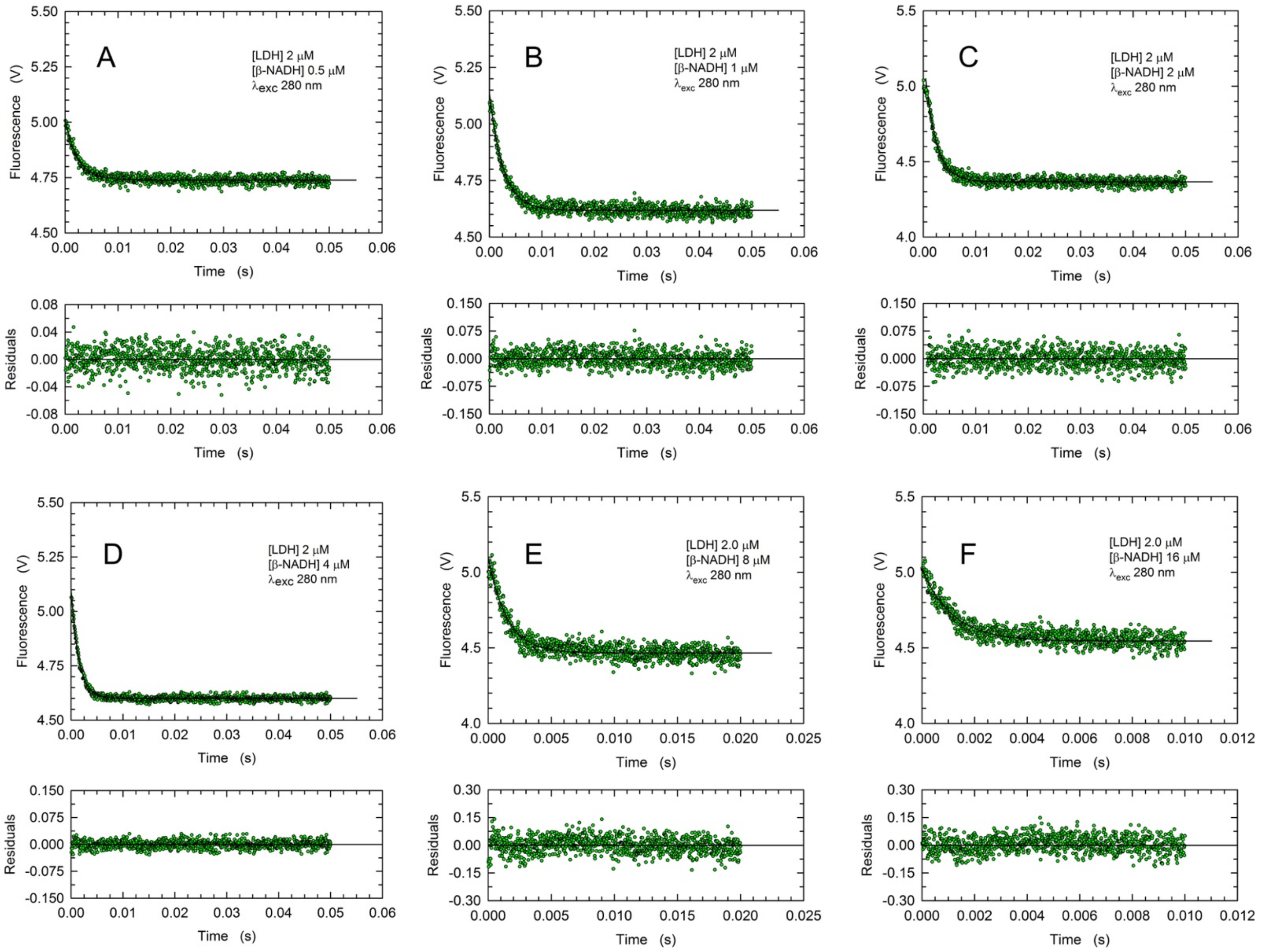

Figure S1. Kinetics of the fluorescence changes (observed over 50, 20, or 10 ms) induced by mixing 4 μM rabbit muscle lactate dehydrogenase with β-NADH at different concentrations. (A-F) Stopped-flow assays were performed at 20 °C by filling the enzyme syringe with 4 μM rabbit LDH-A and the cofactor syringe with β-NADH at concentrations ranging from 1 to 32 μM. Both enzyme and cofactor were buffered with 50 mM Tris-HCl, pH 7.5, and their final concentrations obtained after mixing were as indicated (A-F). To determine the fluorescence of enzyme tryptophanes, samples were excited at 280 nm, and the emission was detected using a longpass filter. The continuous lines represent the best fit of a single exponential equation to the experimental observations.

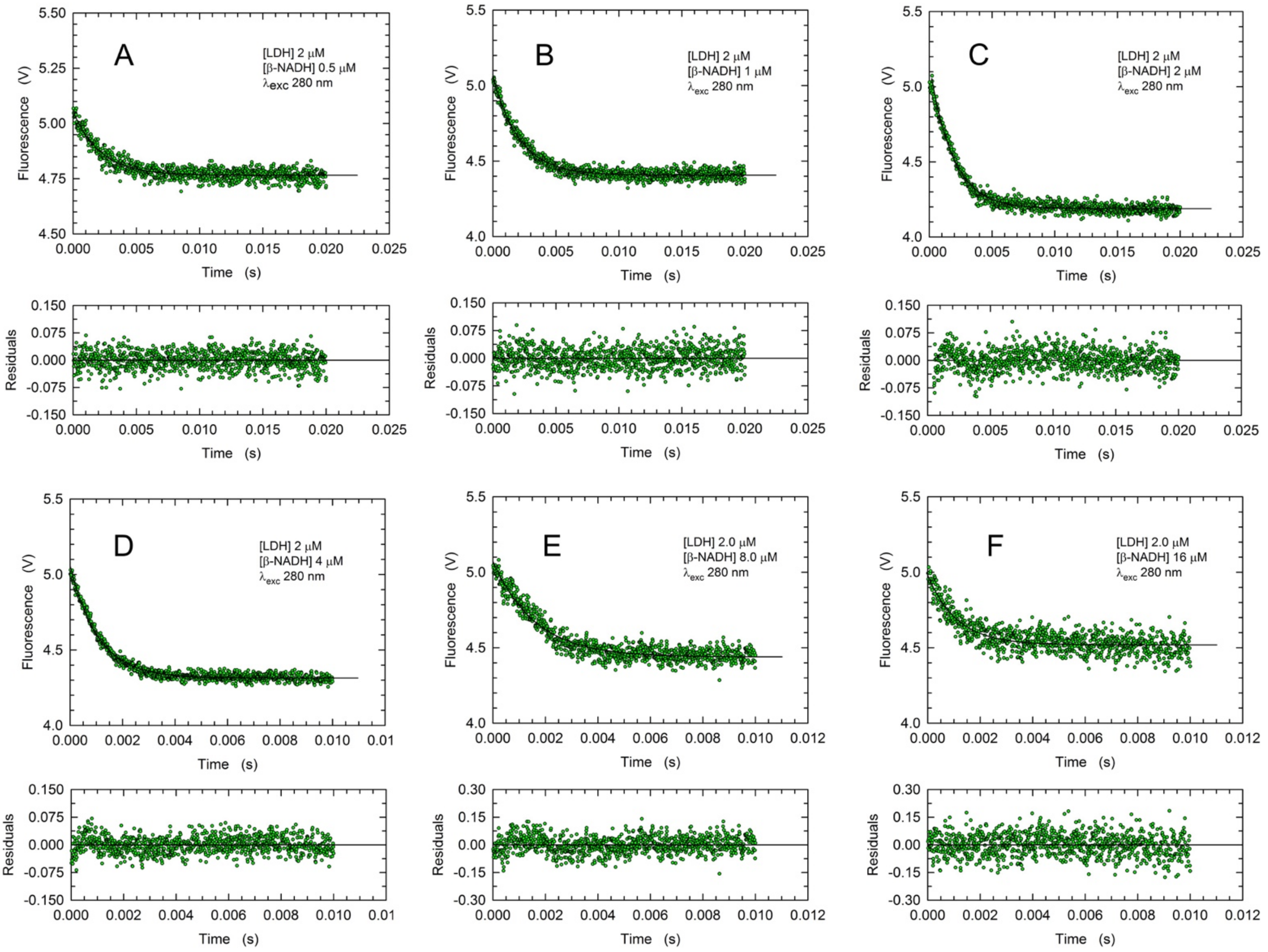

Figure S2. Kinetics of the fluorescence changes (observed over 20 or 10 ms) induced by mixing 4 µM rabbit muscle lactate dehydrogenase with β-NADH at different concentrations.

(A-F) Stopped-flow assays were performed at 20 °C by filling the enzyme syringe with 4 µM rabbit LDH-A and the cofactor syringe with β-NADH at concentrations ranging from 1 to 32 µM. Both enzyme and cofactor were buffered with 50 mM Tris-HCl, pH 7.5, and their final concentrations obtained after mixing were as indicated (A-F). To determine the fluorescence of enzyme tryptophanes, samples were excited at 280 nm, and the emission was detected using a longpass filter. The continuous lines represent the best fit of a single exponential equation to the experimental observations.

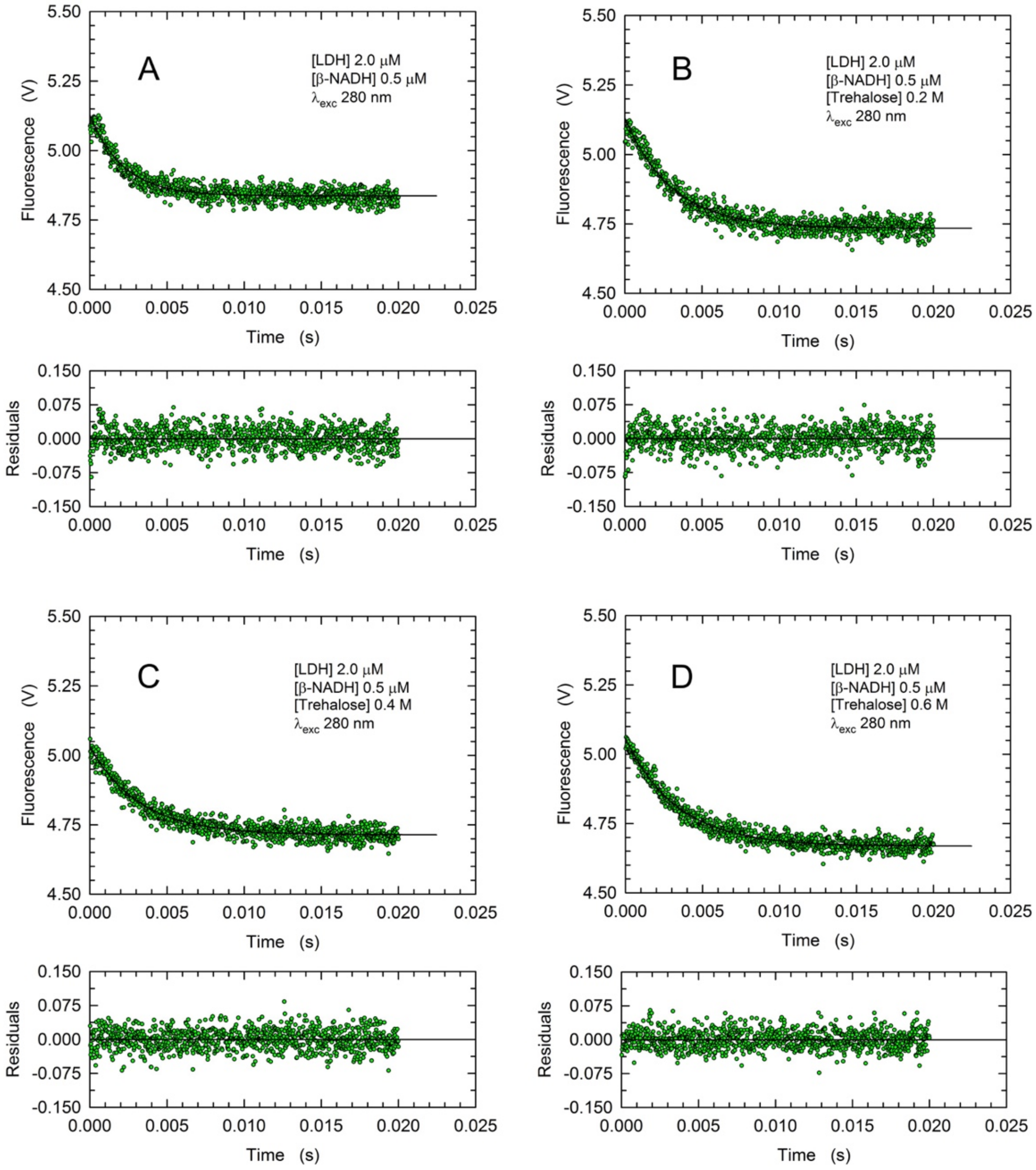

Figure S3. Effect of trehalose on the kinetics (observed over 20 ms) of the fluorescence changes induced by mixing 4 μM rabbit muscle lactate dehydrogenase with 1 μM β-NADH.

(A-D) Stopped-flow assays were performed at 20 °C by filling the enzyme syringe with 4 μM rabbit LDH-A and the cofactor syringe with 1 μM β-NADH, in the absence (A) or in the presence of 0.2 (B), 0.4 (C), or 0.6 (D) M trehalose, respectively. Both enzyme and cofactor were buffered with 50 mM Tris-HCl, pH 7.5. To determine the fluorescence of enzyme tryptophanes, samples were excited at 280 nm, and the emission was detected using a longpass filter. The continuous lines represent the best fit of a single exponential equation to the experimental observations.

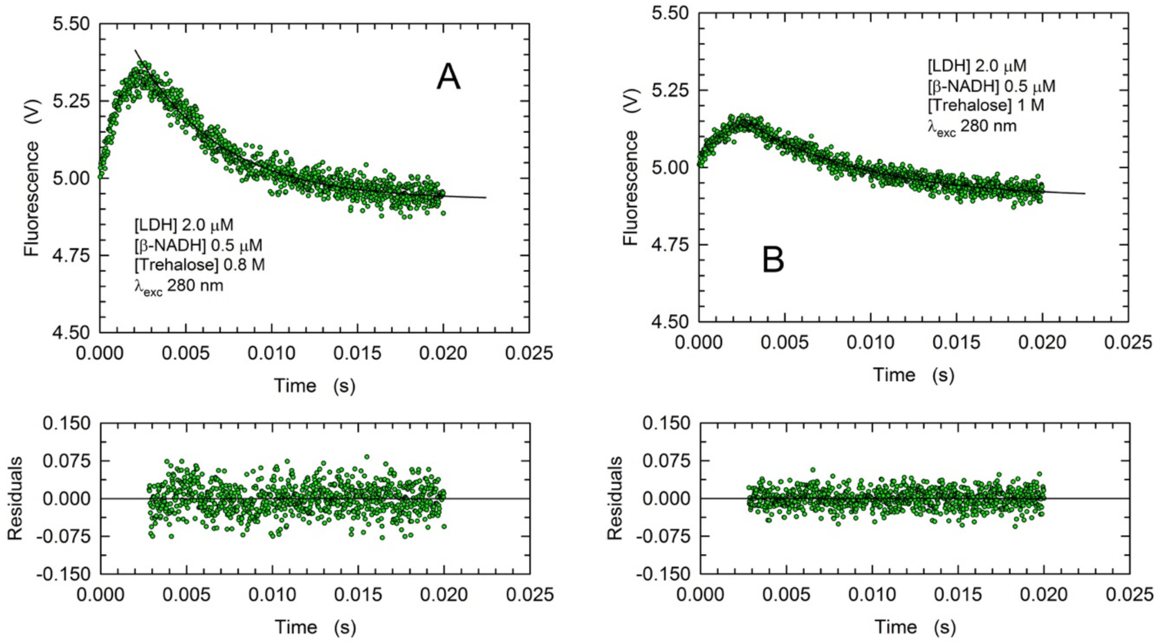

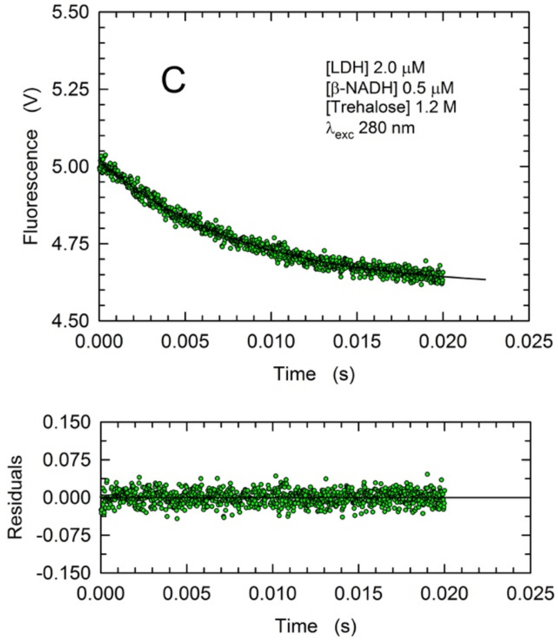

D

[Trehalose] (M)	Observation time (ms)	k_{obs} (s^{-1})	Amplitude (mV)
0	20	469±12	293±5
0.2	20	328±6	398±4
0.4	20	312±6	317±4
0.6	20	299±5	388±3
0.8	20	205±5	416±4
1.0	20	144±5	247±3
1.2	20	123±2	413±2

Figure S4. Effect of trehalose on the kinetics (observed over 20 ms) of the fluorescence changes induced by mixing 4 μM rabbit muscle lactate dehydrogenase with 1 μM β-NADH.
(A-C) Stopped-flow assays were performed at 20 °C by filling the enzyme syringe with 4 μM rabbit LDH-A and the cofactor syringe with 1 μM β-NADH, in the presence of 0.8 (A), 1 (B), or 1.2 (C) M trehalose, respectively. Both enzyme and cofactor were buffered with 50 mM Tris-HCl, pH 7.5. To determine the fluorescence of enzyme tryptophanes, samples were excited at 280 nm, and the emission was detected using a longpass filter. The continuous lines represent the best fit of a single exponential equation to the experimental observations. The estimated values for the k_{obs} and the amplitude of each reaction shown in Figures S3 and S4 are reported in D.

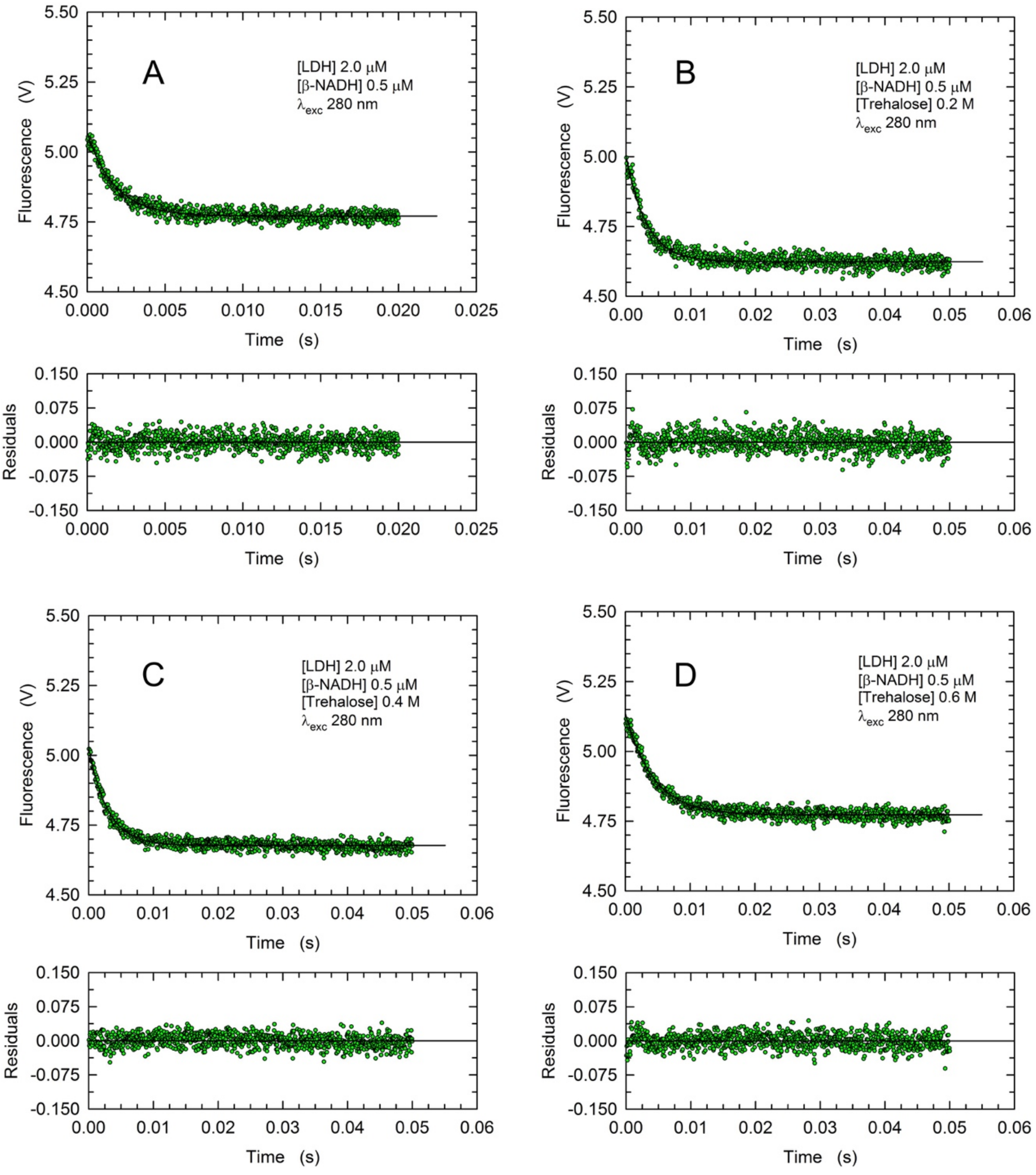

Figure S5. Effect of trehalose on the kinetics (observed over 20 or 50 ms) of the fluorescence changes induced by mixing 4 μM rabbit muscle lactate dehydrogenase with 1 μM β-NADH.
(A-D) Stopped-flow assays were performed at 20 °C by filling the enzyme syringe with 4 μM rabbit LDH-A and the cofactor syringe with 1 μM β-NADH, in the absence (A) or in the presence of 0.2 (B), 0.4 (C), or 0.6 (D) M trehalose, respectively. Both enzyme and cofactor were buffered with 50 mM Tris-HCl, pH 7.5. To determine the fluorescence of enzyme tryptophanes, samples were excited at 280 nm, and the emission was detected using a longpass filter. The continuous lines represent the best fit of a single exponential equation to the experimental observations.

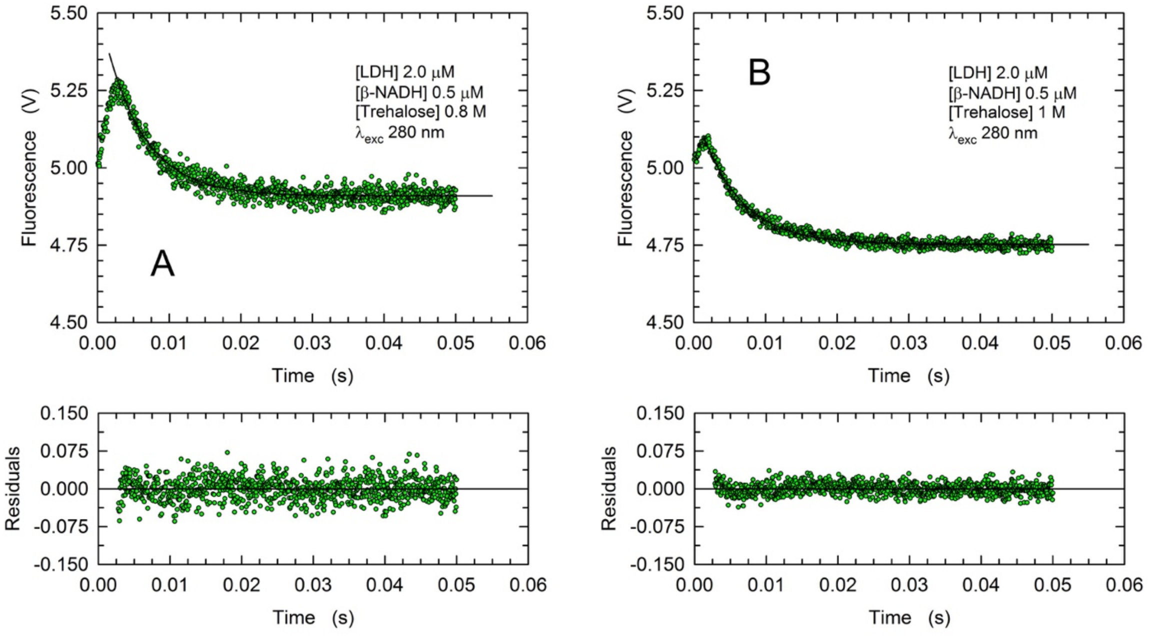

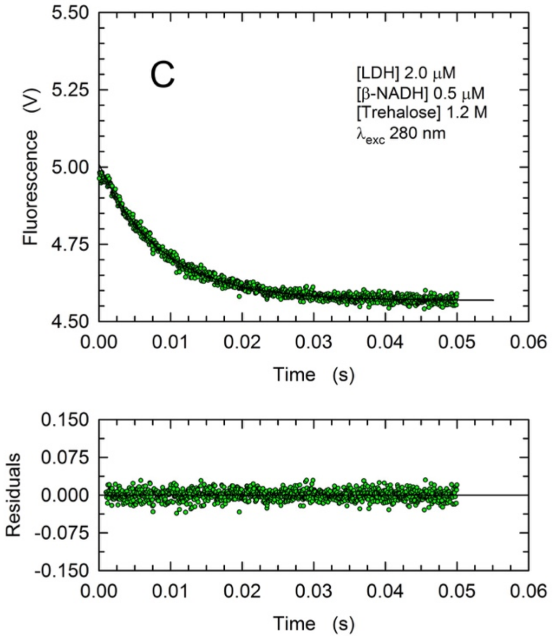

D

[Trehalose] (M)	Observation time (ms)	k_{obs} (s^{-1})	Amplitude (mV)
0	20	560±54	295±3
0.2	50	321±6	380±5
0.4	50	341±6	350±4
0.6	50	232±3	363±3
0.8	50	180±3	375±4
1.0	50	174±2	272±2
1.2	50	115±1	441±2

Figure S6. Effect of trehalose on the kinetics (observed over 50 ms) of the fluorescence changes induced by mixing 4 μM rabbit muscle lactate dehydrogenase with 1 μM β-NADH.
(A-C) Stopped-flow assays were performed at 20 °C by filling the enzyme syringe with 4 μM rabbit LDH-A and the cofactor syringe with 1 μM β-NADH, in the presence of 0.8 (A), 1 (B), or 1.2 (C) M trehalose, respectively. Both enzyme and cofactor were buffered with 50 mM Tris-HCl, pH 7.5. To determine the fluorescence of enzyme tryptophanes, samples were excited at 280 nm, and the emission was detected using a longpass filter. The continuous lines represent the best fit of a single exponential equation to the experimental observations. The estimated values for the k_{obs} and the amplitude of each reaction shown in Figures S5 and S6 are reported in D.

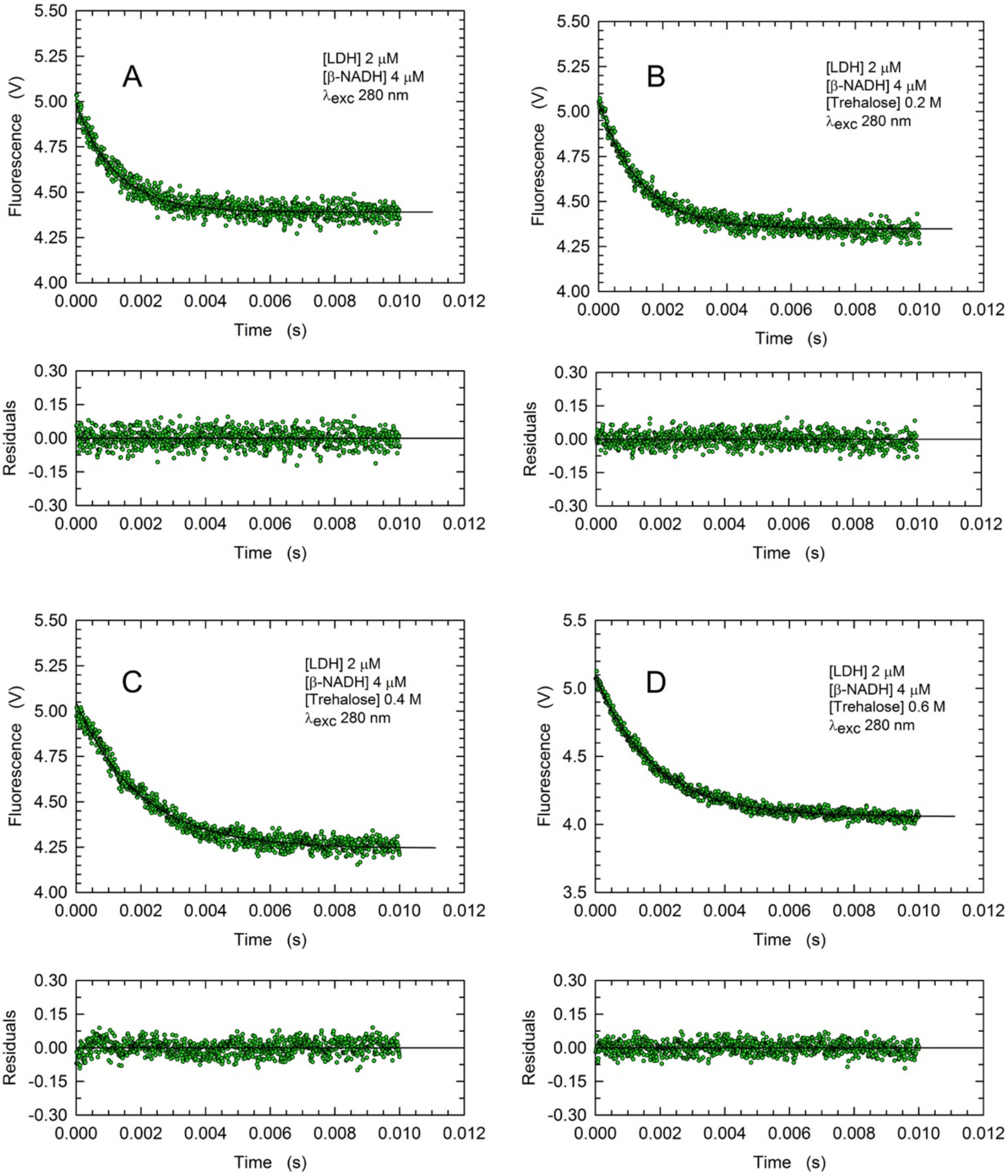

Figure S7. Effect of trehalose on the kinetics (observed over 10 ms) of the fluorescence changes induced by mixing 4 μM rabbit muscle lactate dehydrogenase with 8 μM β-NADH.
(A-D) Stopped-flow assays were performed at 20 °C by filling the enzyme syringe with 4 μM rabbit LDH-A and the cofactor syringe with 8 μM β-NADH, in the absence (A) or in the presence of 0.2 (B), 0.4 (C), or 0.6 (D) M trehalose, respectively. Both enzyme and cofactor were buffered with 50 mM Tris-HCl, pH 7.5. To determine the fluorescence of enzyme tryptophanes, samples were excited at 280 nm, and the emission was detected using a longpass filter. The continuous lines represent the best fit of a single exponential equation to the experimental observations.

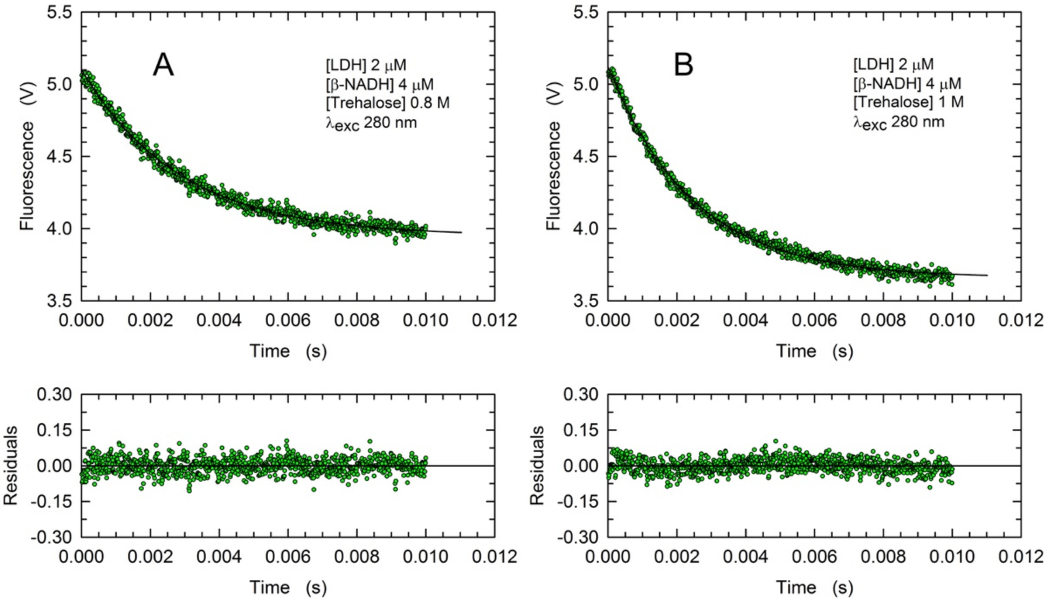

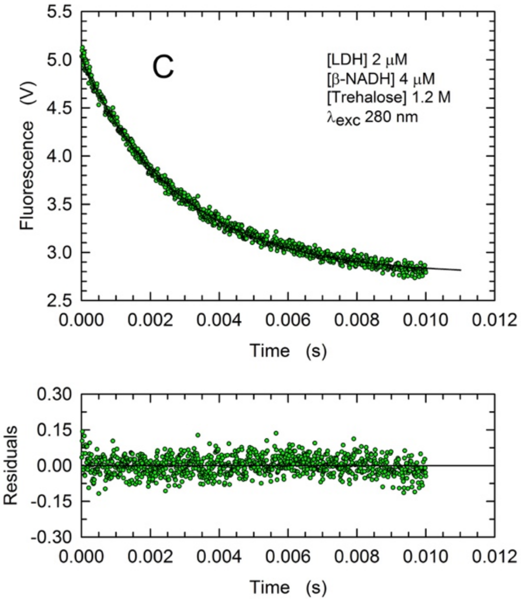

D

[Trehalose] (M)	Observation time (ms)	k_{obs} (s^{-1})	Amplitude (mV)
0	10	812±16	595±7
0.2	10	765±11	707±6
0.4	10	510±6	803±5
0.6	10	558±4	1040±4
0.8	10	350±3	1151±4
1.0	10	406±3	1462±4
1.2	10	350±2	2239±5

Figure S8. Effect of trehalose on the kinetics (observed over 10 ms) of the fluorescence changes induced by mixing 4 µM rabbit muscle lactate dehydrogenase with 8 µM β-NADH.
(A-C) Stopped-flow assays were performed at 20 °C by filling the enzyme syringe with 4 µM rabbit LDH-A and the cofactor syringe with 8 µM β-NADH, in the presence of 0.8 (A), 1 (B), or 1.2 (C) M trehalose, respectively. Both enzyme and cofactor were buffered with 50 mM Tris-HCl, pH 7.5. To determine the fluorescence of enzyme tryptophanes, samples were excited at 280 nm, and the emission was detected using a longpass filter. The continuous lines represent the best fit of a single exponential equation to the experimental observations. The estimated values for the k_{obs} and the amplitude of each reaction shown in Figures S7 and S8 are reported in D.

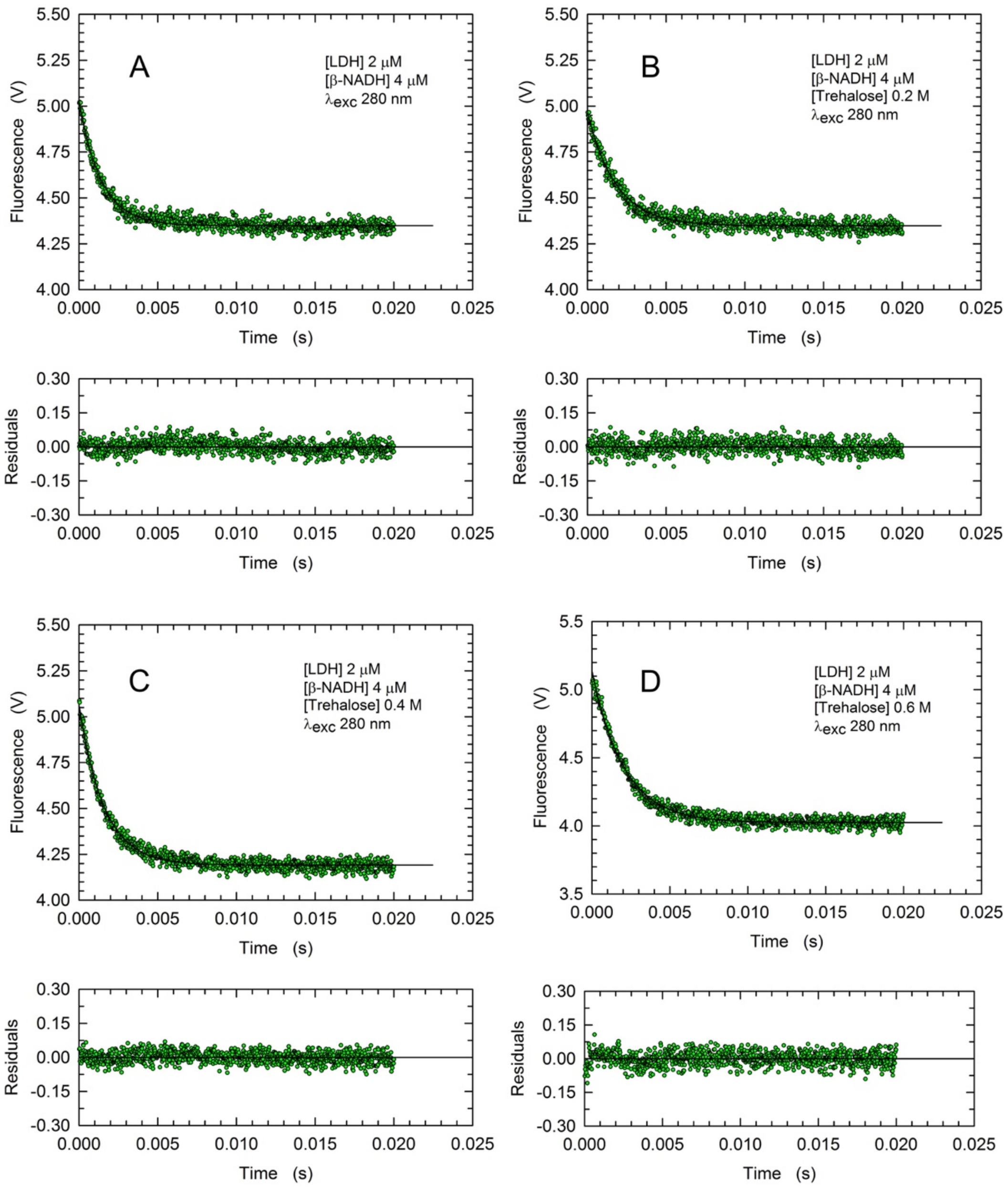

Figure S9. Effect of trehalose on the kinetics (observed over 20 ms) of the fluorescence changes induced by mixing 4 μM rabbit muscle lactate dehydrogenase with 8 μM β-NADH.
(A-D) Stopped-flow assays were performed at 20 °C by filling the enzyme syringe with 4 μM rabbit LDH-A and the cofactor syringe with 8 μM β-NADH, in the absence (A) or in the presence of 0.2 (B), 0.4 (C), or 0.6 (D) M trehalose, respectively. Both enzyme and cofactor were buffered with 50 mM Tris-HCl, pH 7.5. To determine the fluorescence of enzyme tryptophanes, samples were excited at 280 nm, and the emission was detected using a longpass filter. The continuous lines represent the best fit of a single exponential equation to the experimental observations.

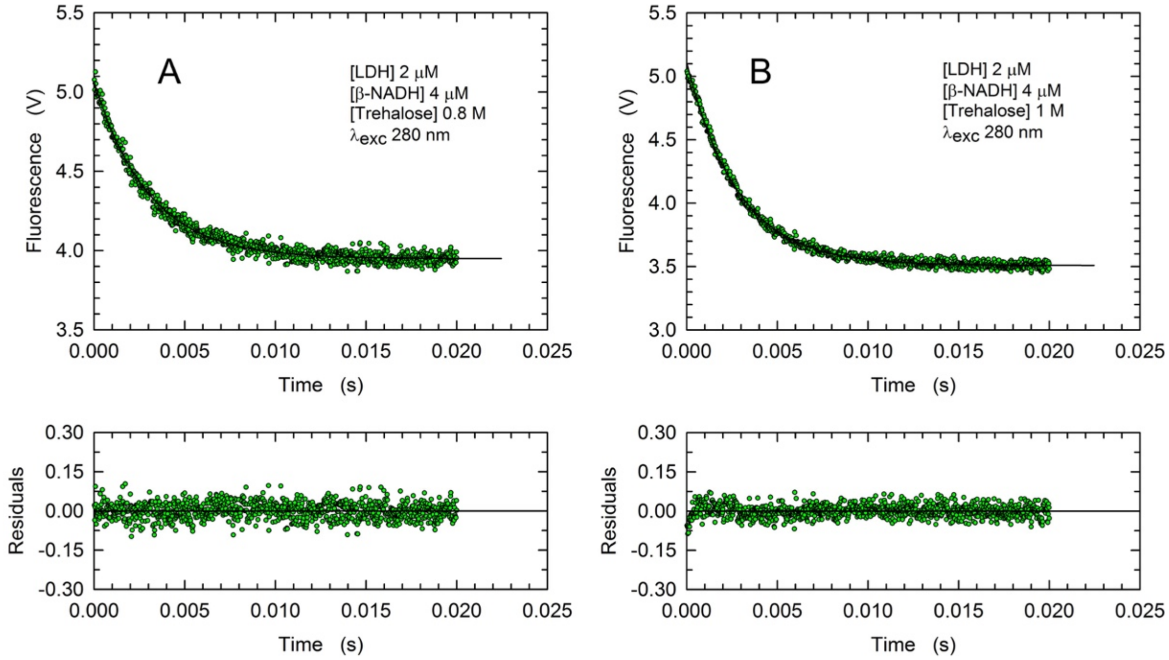

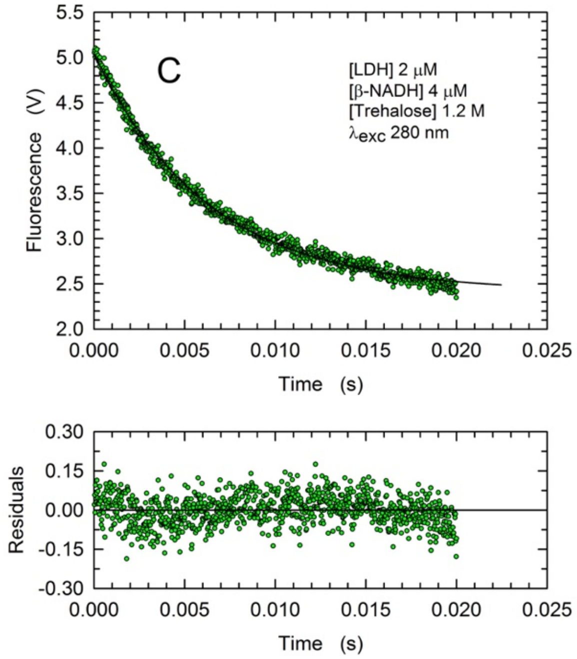

D

[Trehalose] (M)	Observation time (ms)	k_{obs} (s^{-1})	Amplitude (mV)
0	20	712±10	676±7
0.2	20	552±8	618±6
0.4	20	630±6	870±6
0.6	20	491±4	1111±6
0.8	20	332±3	1114±5
1.0	20	355±2	1597±4
1.2	20	159±1	2626±7

Figure S10. Effect of trehalose on the kinetics (observed over 20 ms) of the fluorescence changes induced by mixing 4 μM rabbit muscle lactate dehydrogenase with 8 μM β-NADH.
(A-C) Stopped-flow assays were performed at 20 °C by filling the enzyme syringe with 4 μM rabbit LDH-A and the cofactor syringe with 8 μM β-NADH, in the presence of 0.8 (A), 1 (B), or 1.2 (C) M trehalose, respectively. Both enzyme and cofactor were buffered with 50 mM Tris-HCl, pH 7.5. To determine the fluorescence of enzyme tryptophanes, samples were excited at 280 nm, and the emission was detected using a longpass filter. The continuous lines represent the best fit of a single exponential equation to the experimental observations. The estimated values for the k_{obs} and the amplitude of each reaction shown in Figures S9 and S10 are reported in D

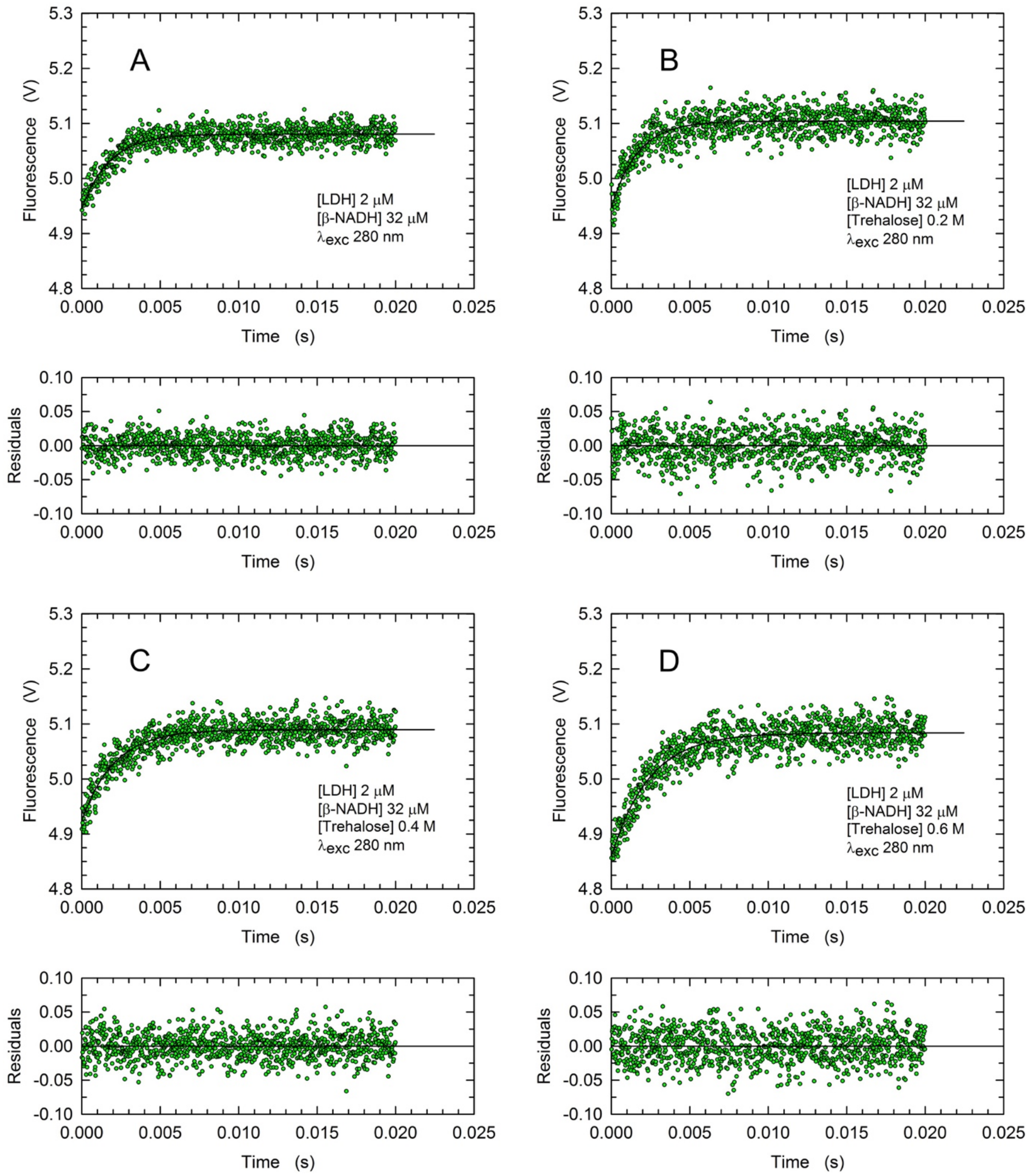

Figure S11. Effect of trehalose on the kinetics (observed over 20 ms) of the fluorescence changes induced by mixing with buffer a solution containing 4 μM rabbit muscle lactate dehydrogenase and 64 μM β-NADH.

(A-D) Stopped-flow assays were performed at 20 °C by filling a syringe with 4 μM rabbit LDH-A and 64 μM β-NADH and a second syringe with only buffer, in the absence (A) or in the presence of 0.2 (B), 0.4 (C), or 0.6 (D) M trehalose, respectively. Both enzyme and cofactor were buffered with 50 mM Tris-HCl, pH 7.5. To determine the fluorescence of enzyme tryptophanes, samples were excited at 280 nm, and the emission was detected using a longpass filter. The continuous lines represent the best fit of a single exponential equation to the experimental observations.

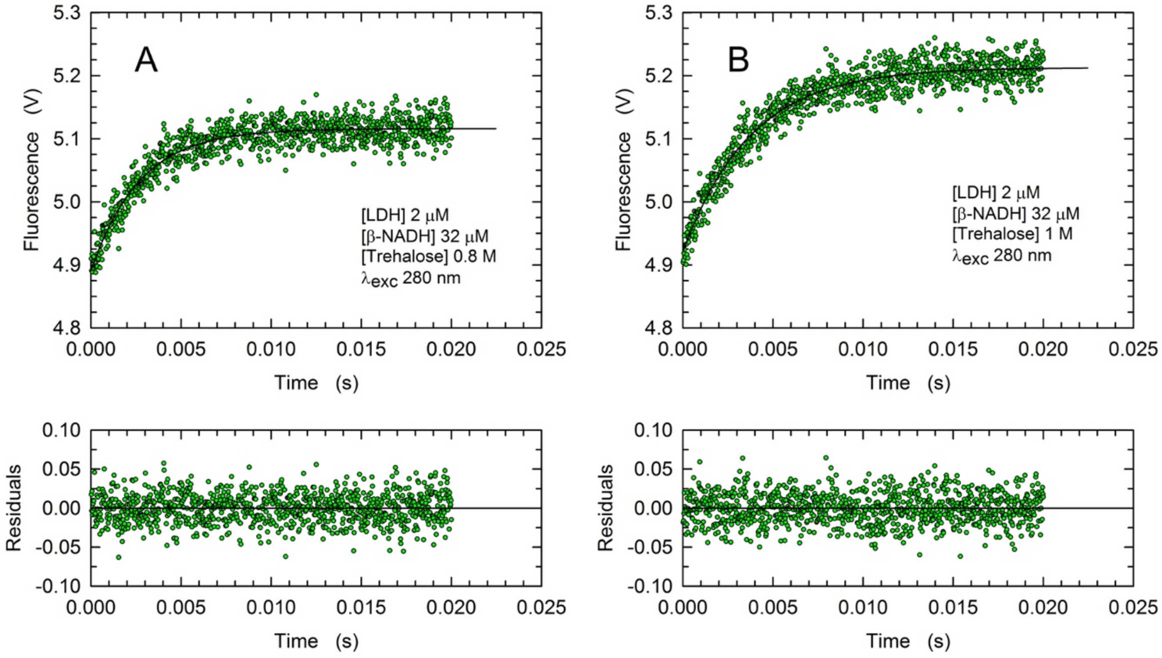

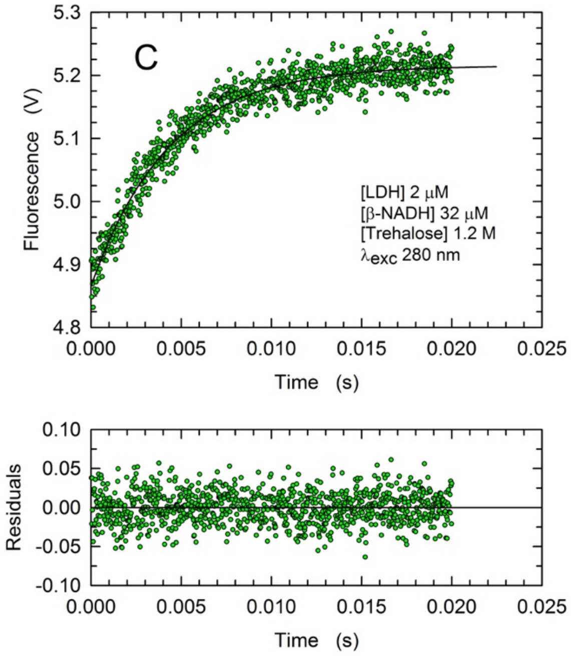

D

[Trehalose] (M)	Observation time (ms)	k_{obs} (s^{-1})	Amplitude (mV)
0	20	573±22	136±3
0.2	20	603±29	157±5
0.4	20	488±18	165±4
0.6	20	416±13	225±4
0.8	20	372±10	225±3
1.0	20	267±6	292±3
1.2	20	230±4	350±3

Figure S12. Effect of trehalose on the kinetics (observed over 20 ms) of the fluorescence changes induced by mixing with buffer a solution containing 4 μM rabbit muscle lactate dehydrogenase and 64 μM β-NADH.

(A-C) Stopped-flow assays were performed at 20 °C by filling a syringe with 4 μM rabbit LDH-A and 64 μM β-NADH and a second syringe with only buffer, in the presence of 0.8 (A), 1 (B), or 1.2 (C) M trehalose, respectively. Both enzyme and cofactor were buffered with 50 mM Tris-HCl, pH 7.5. To determine the fluorescence of enzyme tryptophanes, samples were excited at 280 nm, and the emission was detected using a longpass filter. The continuous lines represent the best fit of a single exponential equation to the experimental observations. The estimated values for the k_{obs} and the amplitude of each reaction shown in Figures S11 and S12 are reported in D.

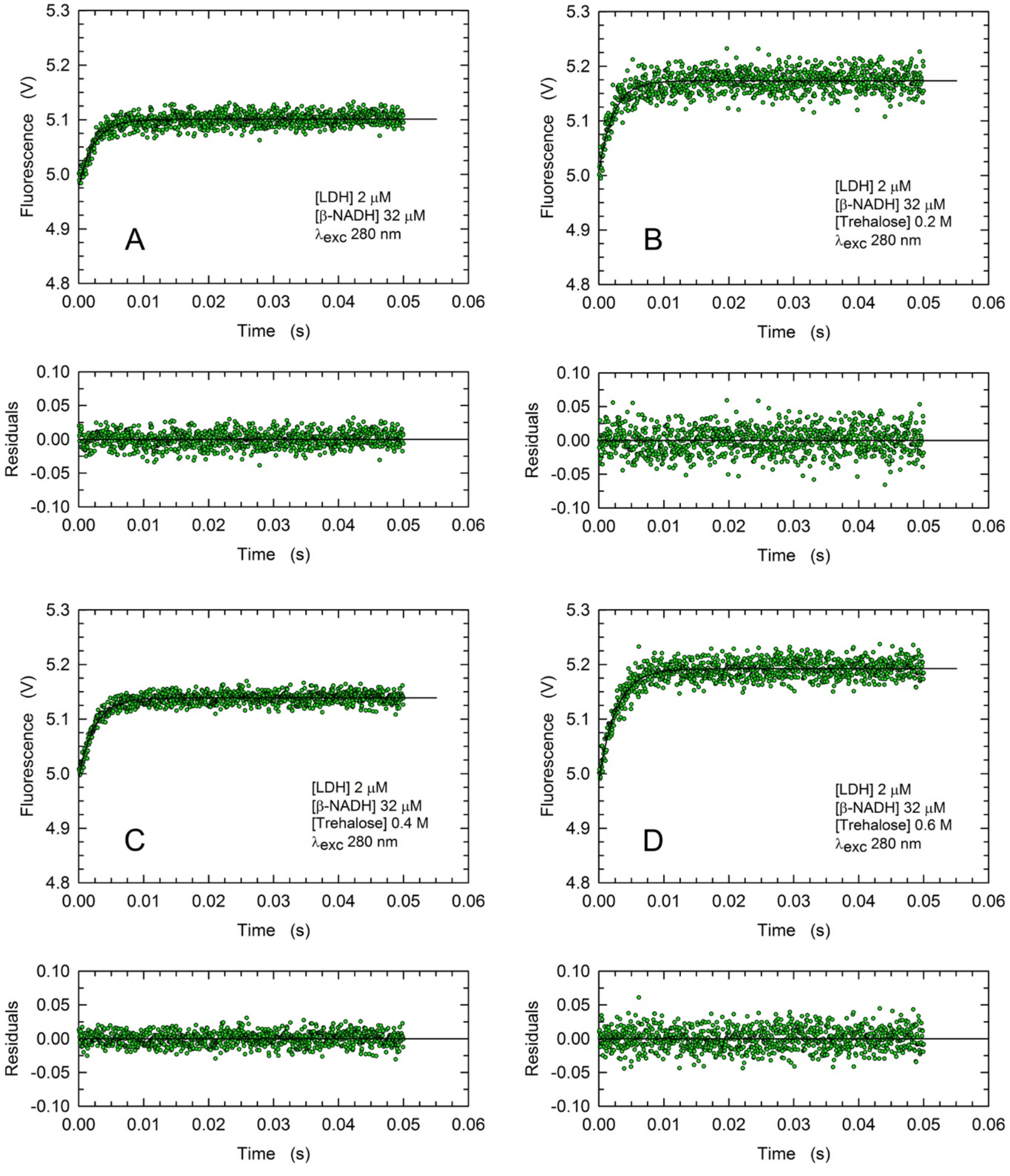

Figure S13. Effect of trehalose on the kinetics (observed over 50 ms) of the fluorescence changes induced by mixing with buffer a solution containing 4 μM rabbit muscle lactate dehydrogenase and 64 μM β-NADH.

(A-D) Stopped-flow assays were performed at 20 °C by filling a syringe with 4 μM rabbit LDH-A and 64 μM β-NADH and a second syringe with only buffer, in the absence (A) or in the presence of 0.2 (B), 0.4 (C), or 0.6 (D) M trehalose, respectively. Both enzyme and cofactor were buffered with 50 mM Tris-HCl, pH 7.5. To determine the fluorescence of enzyme tryptophanes, samples were excited at 280 nm, and the emission was detected using a longpass filter. The continuous lines represent the best fit of a single exponential equation to the experimental observations.

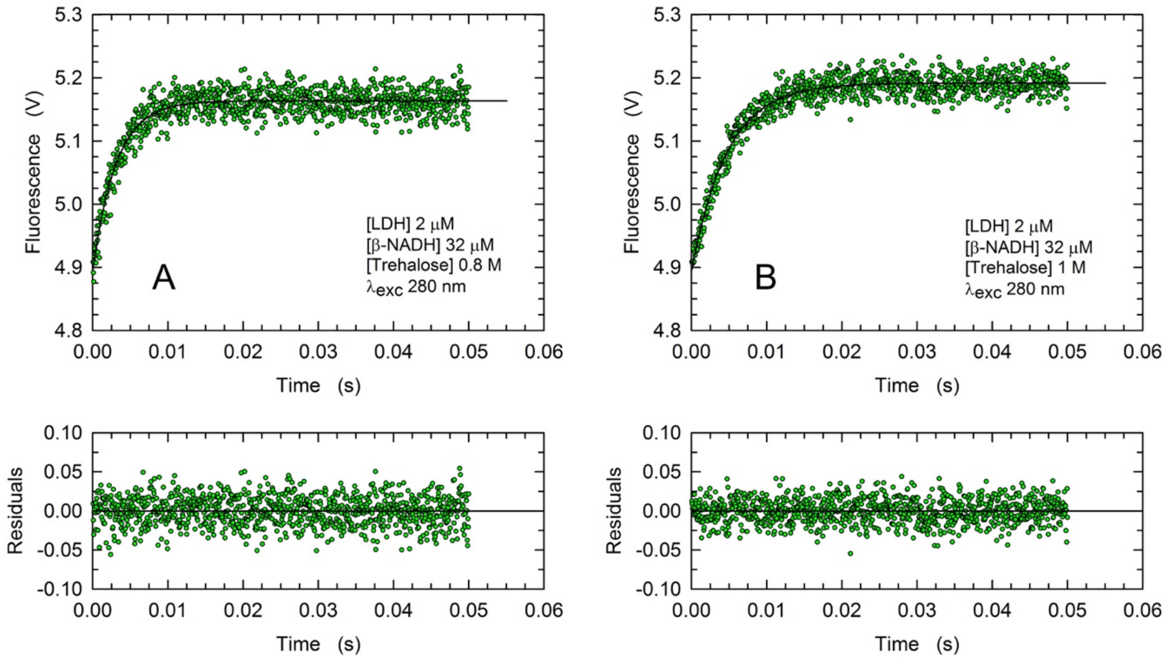

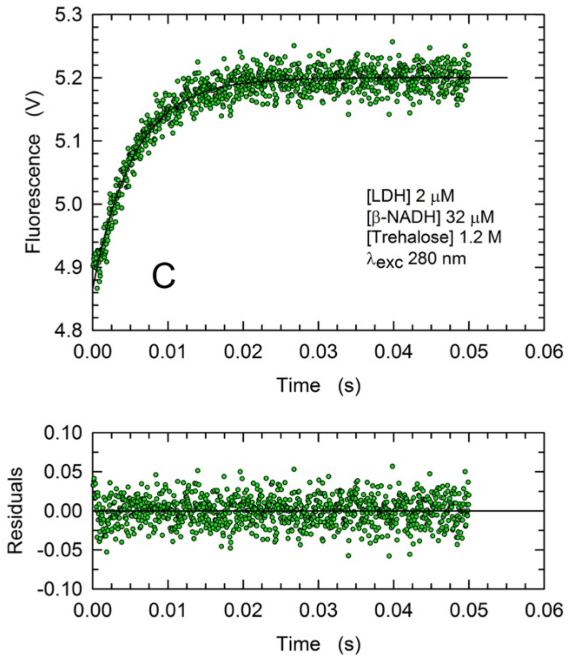

D

[Trehalose] (M)	Observation time (ms)	k_{obs} (s^{-1})	Amplitude (mV)
0	50	417±17	122±3
0.2	50	443±23	165±6
0.4	50	414±12	147±3
0.6	50	372±12	205±4
0.8	50	319±9	269±5
1.0	50	212±4	299±3
1.2	50	181±3	334±4

Figure S14. Effect of trehalose on the kinetics (observed over 50 ms) of the fluorescence changes induced by mixing with buffer a solution containing 4 μM rabbit muscle lactate dehydrogenase and 64 μM β-NADH.

(A-C) Stopped-flow assays were performed at 20 °C by filling a syringe with 4 μM rabbit LDH-A and 64 μM β-NADH and a second syringe with only buffer, in the presence of 0.8 (A), 1 (B), or 1.2 (C) M trehalose, respectively. Both enzyme and cofactor were buffered with 50 mM Tris-HCl, pH 7.5. To determine the fluorescence of enzyme tryptophanes, samples were excited at 280 nm, and the emission was detected using a longpass filter. The continuous lines represent the best fit of a single exponential equation to the experimental observations. The estimated values for the k_{obs} and the amplitude of each reaction shown in Figures S13 and S14 are reported in D.

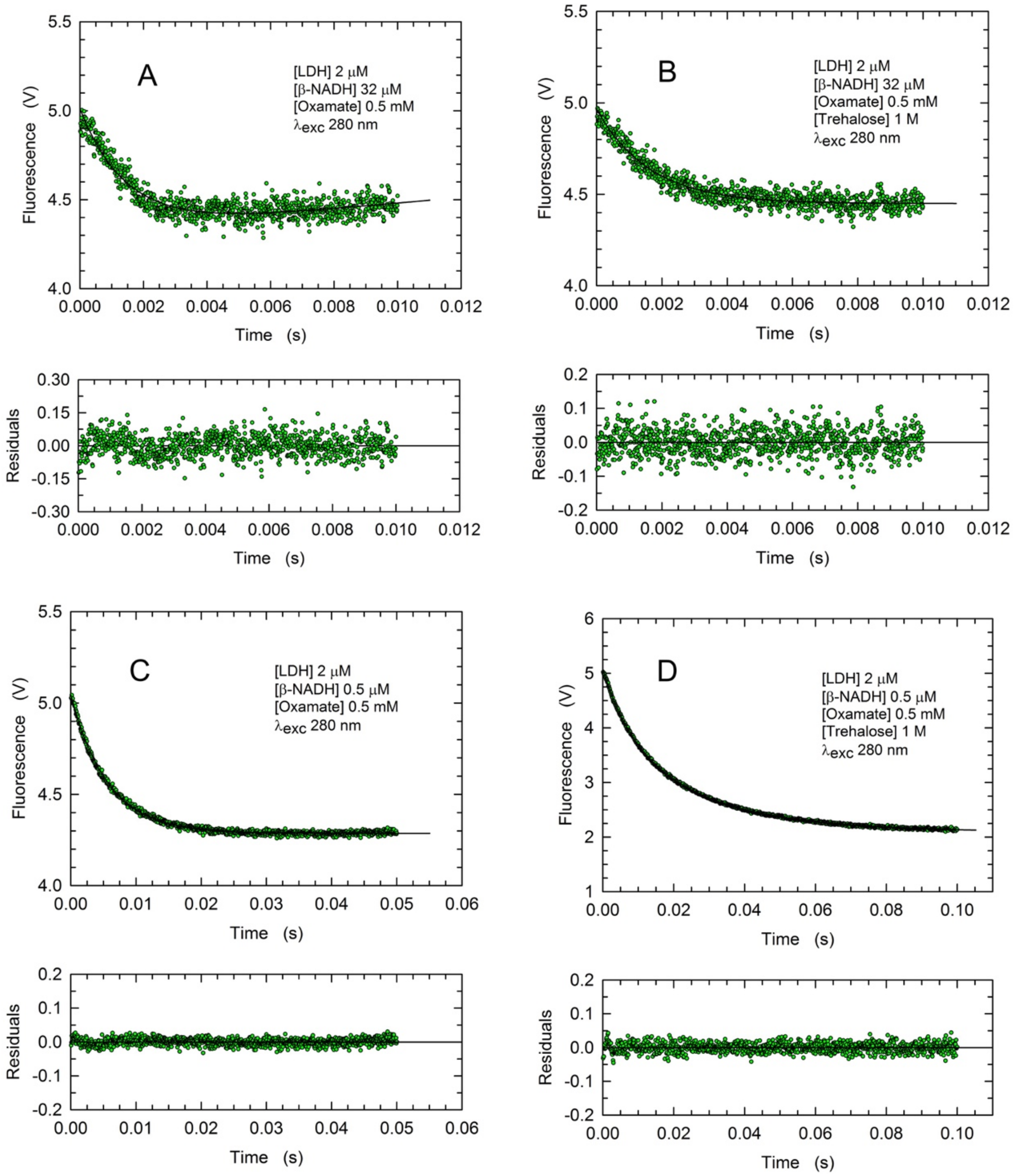

Figure S15. Effect of trehalose on the kinetics of the fluorescence changes induced by mixing with 1 mM oxamate a solution containing 4 μM rabbit muscle lactate dehydrogenase and β-NADH at 64 or 1 μM.

(A,B) Stopped-flow assays were performed at 20 °C by filling a syringe with 4 μM rabbit LDH-A and 64 μM β-NADH and a second syringe with 1 mM oxamate, in the absence (A), or in the presence (B) of 1 M trehalose, respectively. (C,D) Stopped-flow assays were performed at 20 °C by filling a syringe with 4 μM rabbit LDH-A and 1 μM β-NADH and a second syringe with 1 mM oxamate, in the absence (A), or in the presence (B) of 1 M trehalose, respectively. Both enzyme and oxamate solutions were buffered with 50 mM Tris-HCl, pH 7.5. To determine the fluorescence of enzyme tryptophanes, samples were excited at 280 nm, and the emission was detected using a longpass filter. The continuous lines represent the best fit of a single exponential equation to the experimental observations.

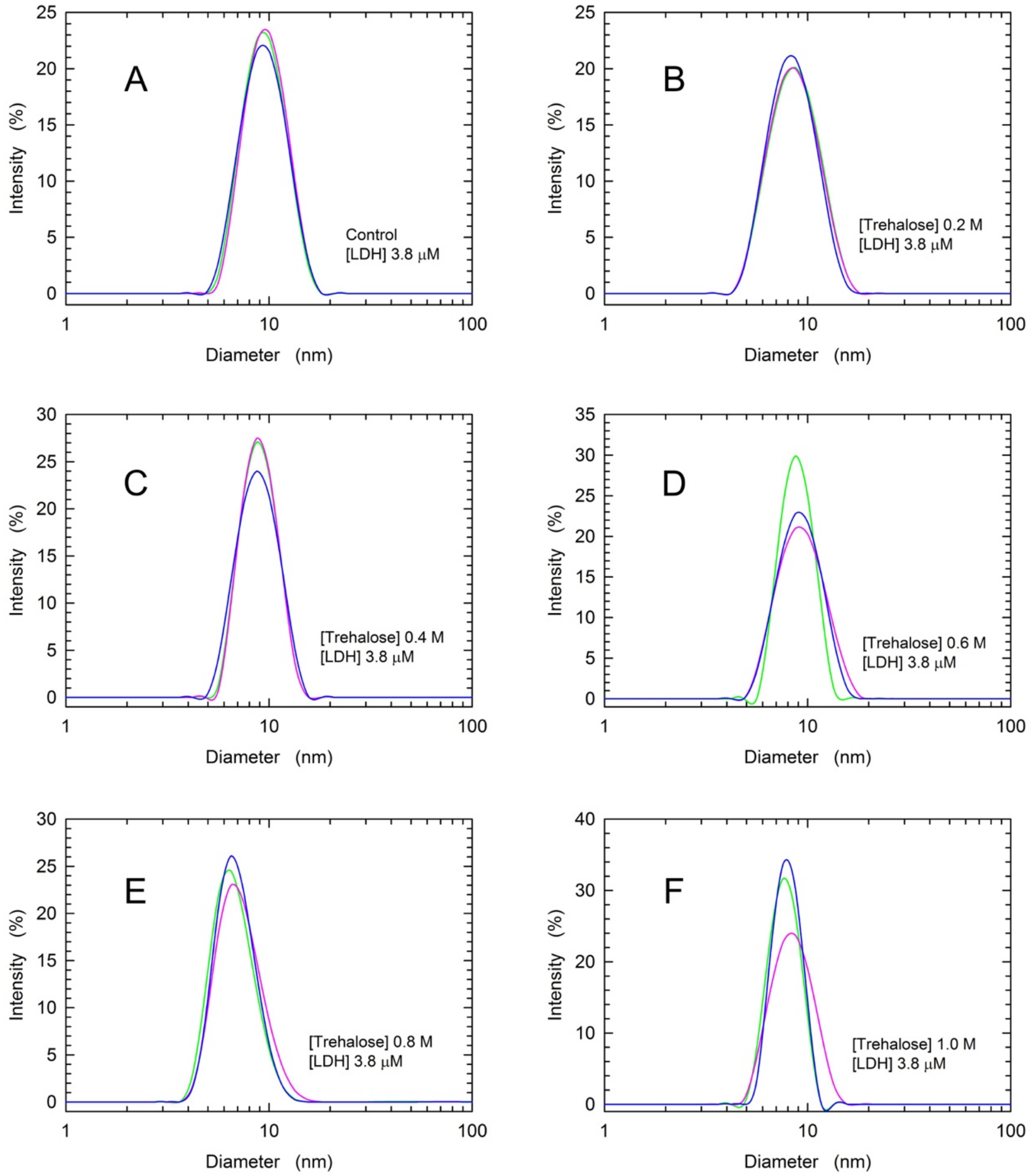

Figure S16. Effect of trehalose on the size of rabbit muscle lactate dehydrogenase.
The diameter of rabbit muscle lactate dehydrogenase was estimated by Dynamic Light Scattering performed with solutions containing 3.8 μM enzyme (concentration of subunits) and poised at 20 °C, in the absence or in the presence of trehalose at the indicated concentrations. All the analyzed samples were buffered using 50 mM Tris-HCl, pH 7.5.

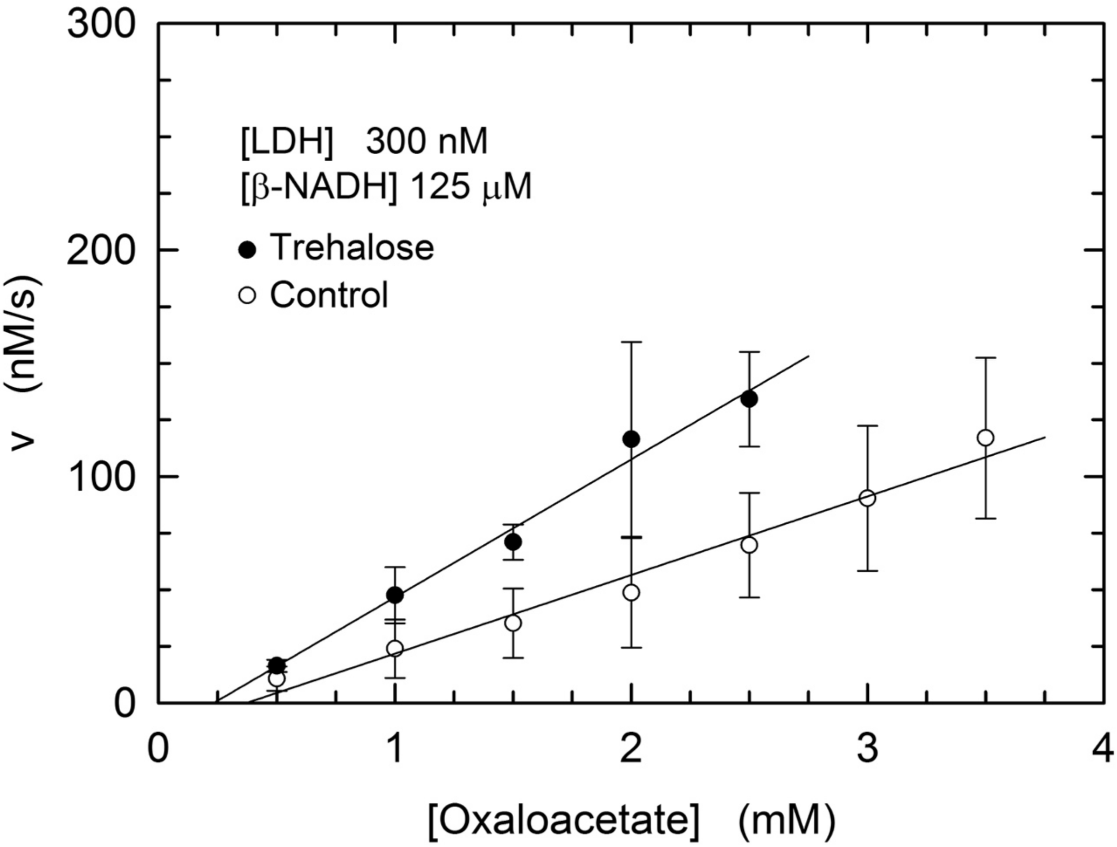

Figure S17. Kinetics of oxaloacetate reduction catalyzed by rbLDH in the absence or in the presence of 1 M trehalose.

Initial velocity of β-NADH oxidation as a function of oxaloacetate concentration in reaction mixtures containing 300 nM rbLDH and 125 μM of the redox cofactor, in the absence (empty circles) or in the presence (filled circles) of 1 M trehalose. All the assays were performed at 20 °C, pH 7.5 (50 mM Tris-HCl). The data shown here are also reported in Figure 8. The continuous lines represent the best fit of a linear equation to the experimental observations. The slopes accordingly determined are equal to 34.7±2.5 and 60.9±4.2 nM•s^{-1}•mM^{-1} for the sample devoid or containing trehalose, respectively.

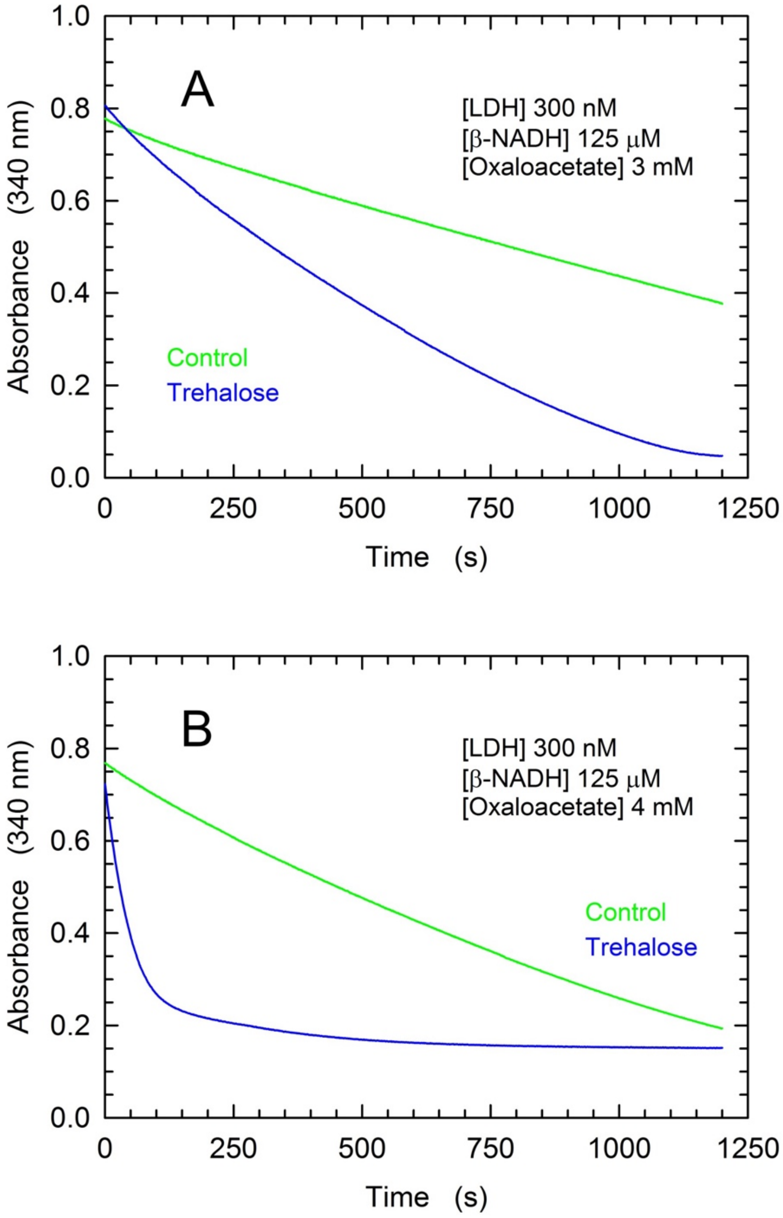

Figure S18. Kinetics of oxaloacetate reduction catalyzed by human lactate dehydrogenase in the absence or in the presence of 1 M trehalose.
(A,B) Kinetics of β-NADH oxidation catalyzed by human LDH-A at the expense of 3 or 4 mM oxaloacetate (A and B, respectively) under the indicated conditions and in the absence (green lines) or in the presence (blue lines) of 1 M trehalose.
